# Mechanisms of Gut Microbiota-Derived Metabolites in Treating Hyperuricemia: Natural Products as Interventions

**DOI:** 10.3390/molecules31142421

**Published:** 2026-07-10

**Authors:** Wenyi Gu, Jianbin Liu, Jae Bin Choi, Kavsar Alim, Siyu Ma, Diliaise Dawuti, Yu Xu, Hongxi Xu

**Affiliations:** 1 School of Pharmacy, Shanghai University of Traditional Chinese Medicine, Shanghai 201203, China; 2 Department of Biological Sciences, College of Humanities and Sciences, National University of Singapore, Singapore 119077, Singapore; 3 Biomedical Sciences and Chinese Medicine, School of Biological Sciences, Nanyang Technological University, Singapore 637551, Singapore

**Keywords:** natural products, hyperuricemia, gut microbiota-derived metabolites, inflammation

## Abstract

Emerging evidence links gut microbiota (GM) dysbiosis to hyperuricemia (HUA). The GM plays a critical role in regulating host health and homeostasis by producing a diverse array of metabolites, including short-chain fatty acids, bile acids and uremic toxins. Dysregulation of the microbial metabolite profile has been implicated in the pathogenesis of HUA. Given the urgent need for green and safe urate-lowering therapies for HUA, recent years have seen an increasing focus on interpreting the ability of natural products to modulate these microbial metabolites. Such interventions enhance beneficial metabolites and suppress uremic toxins, thereby alleviating HUA through coordinated regulation of urate transporters, restoration of intestinal barrier integrity, reprogramming of systemic metabolic disturbances, and inhibition of inflammation via Toll-like receptor 4 (TLR4)/ nuclear factor kappa B (NF-κB), Janus kinase (JAK)/ signal transducer and activator of transcription (STAT), and Phosphatidylinositol-3-kinase (PI3K)/ protein kinase B (AKT) pathways. Furthermore, a comprehensive translational roadmap has been proposed, grounded in a critical appraisal of current trial limitations. Overall, this review consolidates evidence for the protective effects of natural products against HUA and related comorbidities, with an emphasis on GM-derived metabolites, aiming to expand clinical applications and provide insights for future studies.

## 1. Introduction

As an intricate microecosystem, the gut microbiota (GM) is regarded as the “second genome” of the body. Alterations in its structure and composition are closely related to the occurrence of hyperuricemia (HUA), a metabolic disease characterized by disturbed uric acid (UA) metabolism [1]. Causal mechanisms may involve the synthesis of microbiota-derived active metabolites, including short-chain fatty acids (SCFAs), bile acids (BAs) and uremic toxins [2]. These metabolites exert both paracrine and endocrine effects, thereby profoundly influencing local and systemic conditions in the context of HUA-induced intestinal disorders. Among them, SCFAs generated by microbial fermentation and secondary BAs produced by BA conversion help maintain UA homeostasis and act as anti-inflammatory mediators, whereas uremic toxins are often considered as risk factors for renal injury [3,4]. Furthermore, the employment of 16S rRNA sequencing and metabolomics has provided increasing evidence for understanding host–microbiota interactions through the collection of preclinical and clinical data in metabolic diseases [5]. The existing literature supports that changes in GM-derived metabolites are correlated with alterations in amino acid, lipid and glucose levels during UA dysregulation [6]. Therefore, intestinal handling of UA by GM-derived metabolites may represent a microbiome–metabolite–organ axis underlying HUA.

Urate-lowering therapies are currently utilized as first-line drugs for managing HUA symptoms. Animal studies have shown that these therapies promote the growth of beneficial gut bacteria, including *Bifidobacterium* and *Collinsella*, while reducing the relative abundance of harmful pathogens such as *Adlercreutzia* and *Anaerostipes* in order to improve UA consumption [7]. In addition, clinical evidence has indicated that GM function following such treatments exhibits high potential for facilitating carbohydrate metabolism [8]. However, these drugs may cause major adverse reactions that damage liver and renal function, in addition to mobility impairment and gastrointestinal discomfort. Therefore, the pressing need for safe and effective treatments for HUA and associated comorbidities is of great importance and highly justified. Natural products—including phytochemicals (e.g., flavonoids, polyphenols and polysaccharides) in herbal medicine and dietary fiber—can intervene in imbalanced UA metabolism by modulation of GM taxa such as *Lactobacillus* and *Akkermansia*. Moreover, lines of experimental evidence have highlighted their potential of regulating GM and its metabolites, as they participate in improvements in GM dysbiosis, metabolite compositions, intestinal integrity, host metabolic changes and immunity [9,10]. Therefore, we uniquely positioned these GM-derived metabolites as the functional hub that mechanistically links natural product interventions to urate homeostasis. Specially, we pointed out that systemic metabolites influenced by natural products play a critical role in the development of HUA. Furthermore, we provide a comprehensive clinical translation roadmap with tabulated trial evidence, offering a new “metabolite-centric” paradigm to guide future mechanistic and translational studies.

## 2. Search Strategy and Selection Criteria

A comprehensive literature search was performed in the PubMed and Web of Science databases, covering publications from 2016 to 2026, with particular emphasis on studies published within the past five years. The search strategy employed a combination of the following keywords: “natural products,” “gut microbiota,” “microbial metabolites,” “hyperuricemia,” “intestinal barrier,” “amino acid metabolism,” “lipid metabolism,” “glucose metabolism,” “inflammation,” or “oral bioavailability.” Only peer-reviewed, English-language original research and review articles that addressed mechanistic or therapeutic aspects were considered eligible for inclusion. To ensure exhaustive coverage, additional relevant references were retrieved by screening the reference lists of critical reviews and key primary studies.

## 3. Targeting Gut Microecology to Improve UA Homeostasis

**Figure 1:**
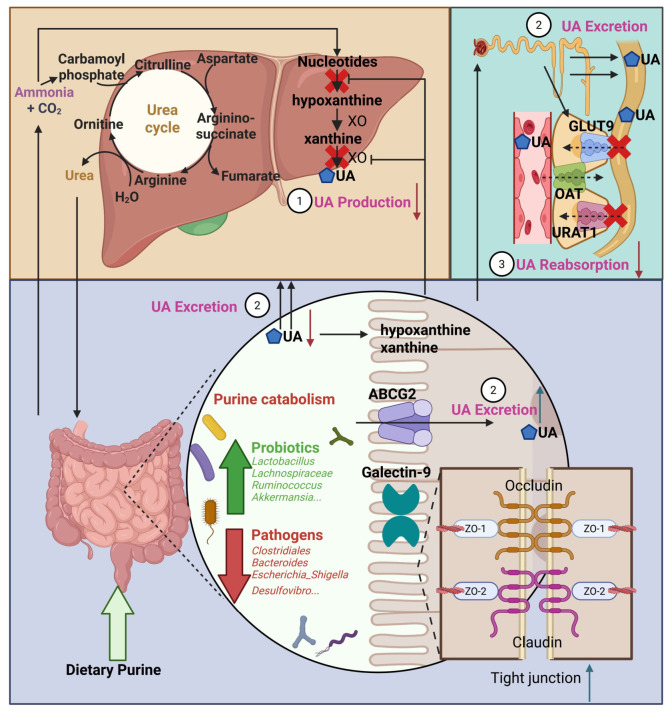
Natural products enhance gut microecology to regulate uric acid (UA) metabolism. During hyperuricemia (HUA), these treatments modify gut microbiota (GM) composition (increasing beneficial bacteria and decreasing pathogens) to inhibit UA overproduction and reabsorption, and promote UA excretion by regulating purine metabolism and urate transporters via the gut–liver–kidney axis. In addition, GM alteration restores intestinal permeability potentially via improving tight junction function, consequently leading to HUA alleviation. ↑, upregulate; ↓, downregulate; ╳, inhibit.

The intestines contribute significantly to the regulation of UA levels in the body, as GM can metabolize UA, accounting for roughly 30% of total UA excretion [11]. Therefore, improving gut microecology with natural products is central to the maintenance of UA homeostasis. Potential mechanisms include gut microbial composition re-modulation and intestinal barrier function enhancement, as shown in Figure 1.

### 3.1. Gut Microbiota Remodeling

The decreased microbial diversity and altered microbial composition known as gut dysbiosis is often caused by the concurrent enrichment of opportunistic bacteria or the relative lack of beneficial taxa and urate-degrading species, consequently contributing to HUA. Therefore, microbial remodeling is crucial to the therapeutic effects of potential anti-HUA interventions. At the phylum level, *Firmicutes* and *Bacteriodetes* constitute roughly 90% of gut flora. During HUA, the ratio of *Firmicutes* to *Bacteriodetes* (F/B ratio) is usually upregulated, whereas it is decreased after phytochemical intervention such as rosmarinic acid (RA) [12]. At the genus level, mounting evidence suggests that their effects on HUA remedies are, at least in part, mediated by enriching the population of beneficial bacteria, such as *Lactobacillus*, *Lachnospiraceae*, and *Ruminococcus*. Naringin, as a natural dietary dihydroflavonoid glycoside, could accurately target intestinal tissues and reshape GM composition by increasing the abundances of probiotics such as *norank_f__Muribaculaceae*, *Lactobacillus*, *Alloprevotella* and *Prevotellaceae_UCG-001* [13]. Polysaccharides from natural sources generally are poor in oral bioavailability, and may display their pharmacological activities by influencing gut flora in ways such as the promotion of growth of *Bacteroides*, *Bifidobacterium*, and *Lactobacillus* to serve as a promising prebiotic to promote intestinal health. A *Viscum coloratum* (Kom.) Nakai-derived neutral polysaccharide, composed of glucose and mannose with a ratio of 1:3.13 M, increased intestinal capacity to degrade UA and promoted the growth of beneficial GM taxa such as *Lactobacillus*, *Peptostreptococcaceae*, and *Ruminococcus*. In addition, these benign GM alterations were positively correlated with activated nuclear factor erythroid 2-related factor 2 (Nrf2)-dependent redox scavenging process and improved the subsequent NOD-like receptor pyrin domain-containing 3 (NLRP3) inflammasome-dependent kidney inflammation and transforming growth factor-beta (TGF-β)-dependent fibrosis in hyperuricemia nephropathy (HN) rats [14].

GM–phytochemical interactions can enhance the production of beneficial bacteria, as well as improve the bioavailability and bioactivity of phytochemicals to improve UA metabolism [15]. Flavonoids are a subset of polyphenols that have a 15-carbon skeleton with a C6–C3–C6 structure and are generally present in glycosylated form in nature. Dietary flavonoids are metabolized in the alimentary canal, with a small portion of undigested flavonoids reaching the colon, where they are further catabolized into smaller units such as aromatic acids, benzoic acids, and phenolic groups with the help of gut microorganisms, thereby elevating their bioavailability in the gut. This process makes GM a key player in the host activity of flavonoids. In addition, structurally, flavonoids react with xanthine oxidase (XO) through hydrophobic binding. Furthermore, their planar structure (hydroxyl benzene ring structure) and the double bonds between C2 and C3 make them more likely to bind to XO. Fermentation by GM can also enhance the bioavailability and efficacy of these active compounds by modulating their metabolic transformation and intestinal absorption into systemic circulation [16]. Lactic bacteria-fermented apple juice helped to increase organic acids (e.g., quinic, lactic, succinic acids), monomeric phenols (e.g., gallic acid, cinnamic acid, vanillic acid) and vitamin Bs, facilitating the alleviation of redox imbalance. Also, this fermentation increased the production of beneficial microorganisms such as o_Lachnospirales, c_Bacteroidia, c_Coriobacteria and o_Oscillospirales in HUA mice [17]. However, this study did not establish a causal link connecting the biotransformation characteristics of organic acids and monomeric phenols, GM alterations and HUA alleviation during fermentation.

### 3.2. Gut Barrier Function Restoration

The barrier system located within the intestines could prevent pathogenic substances in the gut lumen from entering the circulating blood. In the course of HUA, bioactive chemicals improved intestinal barrier integrity and function to counteract HUA [18]. Tripeptide Pro-Glu-Trp derived from whey protein ameliorated HUA-induced intestinal mucosal barrier injury, and promoted the growth of *Ruminococcus*, resulting in an improved intestinal microenvironment. Likewise, peptides from seafish restored the population of *Lactobacillus* and *Blautia* and recovered tricellular tight junction (TJ) function in HUA rats. However, it remained unclear whether these peptides’ improvement on intestinal permeability was correlated with or causal to the enhancement of beneficial GM production or not [19,20]. In addition, increasing the abundance of beneficial GM to improve the metabolic flux of purines, alginate successfully protected gut barrier integrity and function to counteract with HUA [21]. Functioning as the degradation products of alginate, alginate oligosaccharides enjoy an excellent safety profile under both experimental and clinical settings. Notably, “GV-971”, a novel drug comprising sodium oligomannates (derivatives of alginate oligosaccharides), was approved by the National Medical Products Administration (NMPA) of China in 2019. Alginate oligosaccharides also increased butyrate generation to preserve TJ function and promoted probiotic production while eliminating pathogens to accelerate urate excretion [22]. Rhein was highly analogous to febuxostat in structure, serving as an XO inhibitor. Rhein yielded results like the abolishment of UA-induced greater intestinal permeability via decreasing the secretion of fluorescein isothiocyanate–dextran (FITC-dextran) into the bloodstream, reducing the protein expression of Claudin-1 and E-cadherin, and finally lessening mucus secretion. A fecal microbiota transplant (FMT) study further confirmed that modifications of *Lactobacillus* abundance accounted for its UA downregulation ability in the intestines of rodents [23].

## 4. Gut Microbiota-Derived Metabolites as Metabolic Hubs for HUA

Having established that natural products can modulate gut microecology, it is important to elucidate the mechanistic pathways through which GM remodeling is necessary for UA homeostasis. Moreover, the GM functions as a pivotal metabolic powerhouse that produces an array of bioactive molecules, including SCFAs, BAs, and uremic toxins, which serve as critical mediators in host–microbe crosstalk. Upon translocation across the intestinal epithelium, these microbial metabolites profoundly influence host metabolism and immunity. Notably, natural product interventions show potential in reshaping the GM community, contributing to significant alterations in the metabolite landscape, as summarized in Table 1. These metabolite shifts, in turn, result in the amelioration of HUA by suppressing urate biosynthesis, promoting renal and intestinal urate excretion, and suppressing inflammatory responses.

### 4.1. Gut Microbiota-Mediated UA Metabolism: From Synthesis to Excretion

#### 4.1.1. Purine Degradation

Nucleotides are metabolized into purines and pyrimidines; thereafter, purines, as the sources of UA, are transformed into UA in the liver. XO is a critical enzyme in UA synthesis by converting hypoxanthine (HX) to xanthine and ultimately into UA. GM is a major purine reservoir that produces nucleotides in the intestinal mucosa. The duodenum serves as the primary absorption site for dietary purine absorption. However, undigested purines reached the colon, where they are further degraded by the GM [24]. The numerical predominance of the GM over intestinal epithelial cells underscores their potential to compensate for the absence of human oxygen-dependent urate oxidase (Uox), an enzyme decomposing UA to allantoin and H_2_O_2_, through purine-degrading bacteria for UA elimination. The majority of these GMs are anaerobic and widely contain gene clusters (*dpaL*, *hydA*, *ssnA*, *ygeY*, and *xdhD*), such as *Bacillota*, *Fusobacteriota* and *Pseudomonadota*. They rely on purines for carbon and energy, and metabolize a variety of purines and lower HX levels in the intestine. Consequently, they convert UA to purine precursors (e.g., xanthine) via the 2,8-dioxopurine pathway, thereby facilitating UA clearance [25]. Mounting evidence has revealed that *Lactobacillus*, a lactic acid bacteria, not only demonstrated a heightened capacity to inhibit UA biosynthesis via promotion of hydrolase-mediated degradation of purine nucleosides and inhibition of XO activity, but also promoted the improvement of intestinal UA metabolism through modulation of the urea cycle pathway [26]. Resveratrol is a polyphenol rich in grapes, red wine and berries, which has high membrane permeability and undergoes efficient absorption in the intestines, subsequently being metabolized with the help of the GM into conjugated forms. The improvement in microbial function, especially by increasing *Lactobacillus*, accounted for its anti-HUA effects, which consequently led to the upregulation of purine metabolism, as evidenced by the increase in ureidoglycolate dehydrogenase, allantoinase and urease subunits beta and gamma [27]. Epigallocatechin gallate (EGCG), the main polyphenol in green tea, induced modifications in the populations of *Bifidobacterium* and *Faecalibaculum*, promoting the increase in the Prostaglandin E2 (PGE2) level in the cecum and purine-related differentially expressed genes (*Gucy1a1*, *Gucy1b1*, *Gpd1*, *Akap6*, and *Slc25a4*) in the intestinal epithelium to alleviate HUA. However, the observed decrease in the proportion of *Lactobacillus* following EGCG treatment is intriguing and warrants comprehensive investigation to determine whether its probiotic property is strain-specific [28].

#### 4.1.2. Urate Transporter-Associated UA Metabolism

UA metabolism is highly dependent on the activity of urate transporters, as urate is a polar molecule that relies on ion transport channels on the membrane to facilitate secretion and reabsorption in the renal proximal convoluted tubule and intestinal tract (IT). Critical metabolic organs, including the liver and kidney, show a close connection with GM-induced UA metabolism. Due to its low solubility and poor permeability, berberine (BBR) is likely to be accumulated in the gut, enabling potential interactions between BBR and GM. For the modulation of the gut–liver–kidney axis to control UA synthesis and excretion, BBR alleviated potassium oxonate (PO)-induced HUA in mice by suppressing the expression of XO in the liver and urate transporter 1 (URAT1) and glucose transporter 9 (GLUT9) in the kidney. Meanwhile, BBR regulated urate transport in the colon through upregulating ATP-binding cassette transporter G2 (ABCG2) expression and downregulating Galectin-9 expression. Furthermore, BBR increased *Bacteroides*-induced succinic acids, which inhibited adenosine monophosphate deaminase 2 (AMPD_2_) activity to prevent the transformation of adenosine monophosphate into inosine monophosphate in UA production via the gut–liver axis. Notably, this urate-lowering effect was consistently observed in both preclinical models and clinical trials when comparable doses were employed [29]. Oxyberberine (OBB), being an oxidized protoberberine alkaloid and an intestinal metabolite of BBR, reduced serum UA (sUA) levels by increasing the renal activities of organic cation transporter 1/2 (OCT1/2) and organic cation/carnitine transporter 1/2 (OCTN1/2). OBB appreciably activated the gene expression of key enzymes involved in the pentose phosphate pathway (PPP), and inhibited the transcriptional or translational expression of key enzymes in de novo purine biosynthesis (DNPB), as well as the modulated purine salvage pathway (PSP), collectively leading to a reduction in the UA level. The mechanism was intimately associated with favorably harmonizing the gut microflora homeostatic disequilibrium by enriching the abundance of *Lactobacillus* [30]. RA could significantly decrease the abundance of Ruminococcaceae, which was associated with the decrease in metabolites such as flavin adenine dinucleotide and purine. In the meantime, RA reduced UA levels by inhibiting the activities of UA synthase XO and adenosine deaminase (ADA) [31]. Importantly, RA exhibited the highest binding affinity for ABCG2 compared with GLUT9 and URAT1 in the intestine and kidney. Nevertheless, the correlation between GM composition alteration and urate transporter activity remains to be established [32]. Overall, natural products controlled GM-mediated UA biosynthesis via purine degradation promotion, XO inhibition, and a urate transportation system via the gut–liver–kidney axis, representing as a potential therapeutic strategy for HUA therapy, as illustrated in Figure 1.

### 4.2. Alterations in GM-Derived Metabolites

#### 4.2.1. SCFAs

**Figure 2:**
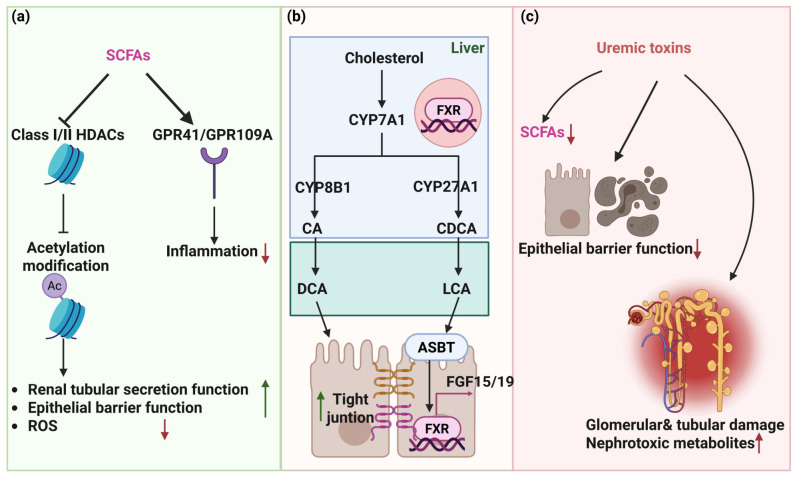
Gut microbiota-derived metabolites have a profound impact on HUA progression. During HUA, GM modifications by natural products may further lead to the alterations in GM-derived metabolites. Among them, short-chain fatty acids (SCFAs) may interact with histone deacetylases (HDACs) and/or G protein-coupled receptors (GPCRs) to improve renal tubular secretion and epithelial barrier function, and inhibit reactive oxygen species (ROS) and inflammation (**a**). Bile acids (BAs), primarily derived from cholesterol, serve as signaling molecules to improve intestinal epithelial function via the gut–liver axis potentially by regulating Farnesoid X Receptor (FXR)-Fibroblast growth factor (FGF)15/19 signaling pathway (**b**). Uremic toxins deteriorated renal and intestinal function, accompanied by reductions in SCFAs contents (**c**). ↑, upregulate; ↓, downregulate.

Metabolomic studies have increasingly uncovered that the altered landscape of inner metabolites constitutes the “active moieties” that mediate the anti-HUA effects of natural products [33], as depicted in Figure 2.

GM-derived SCFAs, including acetic acids (acetates), propionic acids (propionates) and butyric acids (butyrates), are primarily secreted by enterocytes and hepatocytes through the anaerobic fermentation of dietary fibers. However, dietary patterns of HUA patients are quite different from those of healthy individuals, which are lacking in vegetables and abundant in high-purine foods. Based on data from the National Health and Nutrition Examination Survey, a higher dietary index for GM score was significantly associated with a lower likelihood of HUA [34]. As a result, the availability of substrates for microbial fermentation is reduced, indirectly leading to lower intestinal SCFAs production. Therefore, adding dietary fiber intake could serve as a promising candidate for HUA prevention and treatment by inhibiting the digestion and/or absorption of dietary purines. In addition, dietary fiber fostered the growth of SCFAs-producing GM, particularly *Bifidobacterium* and *Prevotella*, to attenuate renal injury via SCFAs-mediated histone deacetylase (HDAC) inhibition and activation of GPR41/GPR109A receptors [35]. Supplementation of insoluble fiber modified GM composition to promote SCFAs generation, contributing to the suppression of URAT1 and GLUT9 activities in the kidneys during HN [36]. However, a systematic review conducted by Vinelli et al. suggested that the effect of dietary fiber on SCFAs seemed to be highly contingent on its dose, origin, and physicochemical structure [37].

Emerging evidence has established that the beneficial effects of natural products are largely mediated by elevated SCFAs concentrations. Natural polyphenols promote SCFAs production by increasing probiotics such as *Lactobacillus* and *Bifidobacterium*. Curcumin is a natural polyphenol that favorably accumulates in the gastrointestinal tract with gut microflora modifications after oral administration. Curcumin treatment led to a more abundant pool in bacteria producing SCFAs, such as *Lactobacillus* and *Ruminococcaceae*, compared with UA nephropathy mice. Yet, the absence of metabolomic analysis in this study leaves the curcumin-induced SCFAs alterations undetermined in this disease context [38]. However, evidence has demonstrated that curcumin elevated systemic SCFAs levels, particularly acetate, which may in turn alleviate renal inflammation under the scenario of chronic kidney disease (CKD) [39]. Following Camellia japonica bee pollen polyphenol treatment, the abundance of beneficial bacteria such as *Lactobacillus* and *Clostridium* was increased, and, accordingly, fecal contents of acetates and butyrates were increased with no significant change in valeric acids in HUA mice. Future studies are warranted to demonstrate the causal link between the remodeling of GM and the relief of inflammation by this polyphenol [40]. In addition, natural flavonoids such as flavonoids in *Paederia scandens* (Lour.) Merrill and green tea increased the pool of *Lactobacillus* and *Bifidobacterium* to produce SCFAs [41]. In accordance with these results, intake of lactic acid probiotic strains such as *Lactobacillus paracasei* N1115 increased the abundance of *Bifidobacterium*, while elevating butyrate levels in the gut, which in turn suppressed serum and hepatic XO activity [42].

SCFAs are also involved in the regulation of the intestinal TJ barrier. For instance, sodium butyrate (200 mg/kg/d) restored intestinal barrier injury, as evidenced by MUC2 and tight junction protein expression against UA overload [43]. Dietary intake of fermentable soluble fibers, namely unmodified guar gum or partially hydrolyzed guar gum (with reduced viscosity to relieve gastric discomfort), both substantially attenuated a colonic barrier defect, partially by increasing bacterial consumption of urea to reduce colonic urea and ammonia concentrations, and elevating levels of both total and individual SCFAs [44]. Coix seed oil enhanced intestinal barrier function in HUA mice by restoring TJ function, which was positively correlated with an expansion of the GM population in *Akkermansia* and *Prevotellaceae_UCG-001*. Moreover, the improved gut microbial metabolism was potentially due to the generation of SCFAs such as acetates, propionates, butyrates, valerates, isobutyrates, and isovalerates [45]. A FMT study revealed that HUA alleviation by oleanolic acid, a natural pentacyclic triterpene, was potentially attributed to GM composition alterations triggered by SCFAs content fulfillment through reinforcing gut barrier integrity and upregulating ABCG2 expression and downregulating GLUT9 expression in the intestines [46]. Clinically, astaxanthin is found to be able to increase the colonization of *Akkermansia* and *Lactobacillus* in the intestine and indole metabolites as well, such as indole-3-lactic acid and indole-3-propionic acid (IPA) [47]. In addition, astaxanthin and *Lactobacillus rhamnosus*, utilizing nanotechnology, exhibited greater intestinal adhesion performance. Meanwhile, this nanosymbiotic enriched health-promoting GM including *Lactobacillus* and *Alloprevotella* to raise acetate, propionate, butyrate, valerate, and isobutyrate levels, and improved TJ integrity to manage HUA symptoms [48]. Nevertheless, whether the SCFAs-mediated enhancement of TJ integrity actually contributes to the therapeutic effects of natural products in HUA remains a critical knowledge gap, given that the existing evidence is largely correlative rather than causative.

#### 4.2.2. Bile Acids

BAs represent another class of GM-metabolized signaling molecules. Produced primarily in the liver from cholesterol, primary BAs are secreted into the duodenum to aid in the absorption of lipids and fat-soluble vitamins. Among them, a small fraction of BAs reaches the colon, where they are metabolized by GMs into intestinal BAs, whereas a great number of BAs are again transported back to the liver after going through bacterial transformation. They act as important signaling molecules to maintain host energy homeostasis and innate immune responses through interactions with nuclear receptors such as Farnesoid X Receptor (FXR) and/or G protein-coupled receptors (GPCRs) [49]. Particularly, intestinal FXR signaling ensures efficient BA efflux through portal circulation while maintaining regulated enterocytic reabsorption, thereby preventing the accumulation of intracellular BAs. GM dysbiosis in HUA may in turn lead to alterations in BA composition, especially the accumulation of primary BAs due to failure of GM metabolization [50]. Zou et al. discovered that *Bifidobacterium* is a probiotic that aids in the conjugation of cholic acid (CA), which makes it easier for 12α-hydroxy BAs to be retained in the enterohepatic circulation. In the ileal of HUA rats, hepatic abundance of CA was positively associated with the levels of total 12α-hydroxy BAs, taurocholic acid (TCA), glycocholic acid (GCA), and taurodeoxycholic acid (TDCA). This process is deeply influenced by the 7α-dehydroxylation of CA to produce DCA through *bai* operon with the interplay of certain species in *Clostridium* and *Bacteroides* [51]. In addition, cumulative evidence has indicated that certain BAs such as CA, chenodeoxycholic acid (CDCA) and tauroursodeoxycholic acid (TUDCA) are potential XO inhibitors to limit hepatic UA production. These results suggest that the regulation of the GM–BA axis influences the progression of HUA.

Natural products effectively modulate BA biosynthesis to limit UA overproduction. Dietary inulin consumption modified the metabolism of microbes with bile salt hydrolases and enzymes that cleave the amide bond of conjugated BAs, resulting in elevated systemic levels of BAs, specifically CA, in an FXR-dependent manner [52]. Due to its glycoside structure, the poor oral bioavailability of secoisolariciresinol diglucoside from flaxseed was enhanced through enzymolysis by the GM into bioactive phytoestrogens, which subsequently facilitate systemic absorption through enterohepatic circulation. Differential intestinal metabolites in this treatment group were enriched in pathways related to purine metabolism, secondary BA biosynthesis and BA secretion. Importantly, the perturbations in these metabolic pathways exhibited a strong correlation with GM compositional dynamics [53]. Hepatic BA profiling revealed that intraperitoneal injection of ELABELA, an endogenous peptide, elevated levels of TCA, TUDCA, and tauro-β-muricholic acid (TβMCA). Furthermore, an in vivo and in vitro study showed that it controlled BA over-synthesis, primarily through suppression of Cytochrome P450 family 27 subfamily A member 1 (CYP27A1), leading to the amelioration of HUA [54]. Dioscin was metabolized into tigogenin by the GM and further enhanced the conversion of cholesterols to BAs via the FXR/ Cytochrome P450 family 7 subfamily A member 1 (CYP7A1) axis in the liver; meanwhile, dioscin increased the level of CA in illeal BAs of mice with HUA-associated atherosclerosis. Clinical data also revealed that one month of dioscin supplementation reduced serum TC and LDL-C levels, suggesting that modulation of cholesterol metabolism was correlated with an HUA remedy [55]. However, a proportion of these studies on natural products did not experimentally distinguish whether these BAs originated from GM metabolism or were exclusively of hepatic origin. In addition, probiotic intake such as *Lacticaseibacillus paracasei* augmented the SCFAs-producing GM community and further led to propionate, butyrate, isovaleric acid, and valeric acid generation. These shifts were positively associated with restored BA metabolism, especially ursodeoxycholic acid 3-sulfonate [56].

BAs, such as CDCA and TUDCA, have been understood to strengthen barrier function, especially by the activation of bile acid receptors including FXR and G protein-coupled bile acid receptor 1 (TGR5). On the other hand, *Clostridium* and *Bacteroides* increase the hydrophobicity of BAs and help the deconjugation of BAs, thus neutralizing excess BAs in the colon to protect colonic mucosa from cytotoxic BA accumulation [57]. Theabrownin from tea extract prevented the accumulation of intestinal BAs, including primary BAs (TCA, GCA, CDCA) and secondary BAs (DCA, TDCA, TUDCA). This further increased intestinal permeability, as shown by the increased levels of ZO-1 and the intestinal mucous barrier’s main contributor, Mucin2, by modulating intestinal FXR signaling [58].

#### 4.2.3. Uremic Toxins

In contrast, the accumulation of GM-derived uremic toxins, including trimethylamine-N-oxide (TMAO), indoxyl sulfate (IS), and p-cresol sulfate (PCS), induces impairment in intestinal homeostasis. TMAO originates from hepatic oxidation of trimethylamine (TMA), which is synthesized by the action of GM in the small intestine on dietary precursors, with betaine, L-carnitine, and choline being the primary sources. These gut microorganisms are normally high in the abundance of *Firmicutes* and low in the abundance of *Bacteroidetes* to produce TMAO via distinct enzymatic pathways (e.g., choline TMA-lyase or carnitine oxygenase/reductase). The reduction in sUA and blood urea nitrogen (BUN) levels were accompanied by the accumulation of serum TMAO concentration, with their production attributed to the enrichment of *Blautia*, *Enterococcus*, and *Faecalibaculum* in renal fibrosis of HN rats. However, these detrimental effects are diminished by chlorogenic acid [59]. In the regards to atherosclerosis, natural products could influence the choline–TMA–TMAO signaling cascade, TMA/TMAO-related lipid metabolism, and vascular endothelial function to remedy the disease state; however, studies on their roles in TMAO in regard to asymptomatic HUA are still lacking. IS and PCS are protein-bound uremic toxins (PBUTs) which had an intimate interplay with GM during their production. They convert from dietary tryptophan (Trp) into indole; indole is further hydroxylated and sulfated by human hepatic cytochrome P450 and sulfotransferase enzymes, ultimately generating IS [60]. PCS is primarily originated from dietary tyrosine, metabolized in the colon by GM and further sulfated in hepatocytes [61]. The intake of inulin enabled the enrichment of *Lachnospiraceae*, *Akkermansia*, *Ruminococcus*, *Bifidobacterium* and a fecal SCFAs pool. Network analysis indicated that inulin-induced SCFAs generation was inversely correlated with the accumulation of IS and PCS in *Uox* knockout (*Uox*-KO) mice [62]. However, the direct impact of inulin on IS and PCS accumulation remains largely unexplored during HUA. Another study showed that in a gut-humanized CKD mice model, intervention with oat-resistant starch ameliorated gut dysbiosis and concurrently reduced IS, PCS, and their precursors indole and p-cresol [63]. Quercetin, especially at the high dose, significantly lowered serum levels of UA, creatinine, and blood urea nitrogen. Also, it reduced the abundance of *Blautia* and *Lachnospiraceae*, which were closely related to the production of uremic toxins, including 3-phenyllactic acid, hippuric acid, and N-acetyl-l-phenylalanine. These findings suggested a potential suppression of microbial phenylalanine metabolism to mitigate renal damage and inflammation, which may contribute to the reduction in nephrotoxic metabolites [64].

The gradual accumulation of GM-derived uremic toxins in the circulating blood is partially attributed to sustained colonic synthesis, compromised renal clearance and increased paracellular translocation across an altered intestinal epithelium as the disease progresses. TMAO lowers the levels of TJ proteins and significantly raises markers of intestinal barrier dysfunction, such as diamine oxidase (DAO), which in turn leads to heightened intestinal permeability. *Panax notoginseng* saponins, especially at high doses of 160 mg/kg, recovered barrier function (as evidenced by decreased DAO level and increased Occludin and ZO-1 concentrations) to downregulate serum TMAO levels, thereby alleviating adenine-induced kidney damage [65]. However, limited studies interpreted the effects of PBUTs on influencing TJ function during HUA, which necessitates further investigation.

#### 4.2.4. Interplay Between Intestinal Urate Transporters and Microbiota-Derived Metabolites

GM-induced metabolite changes participate in the regulation of UA excretion and absorption, especially influencing urate transporter activities. However, as the disease progresses, the adaptive changes in intestinal urate handling that occur when renal urate excretion declines are indicative of the Remote Sensing and Signaling Theory. Accordingly, this theory highlighted the central role of intestinal urate transporters such as (OAT)1/3 and ABCG2 in mediating UA excretion and influencing GM-derived metabolites during HUA [66]. SCFAs improved intestinal ABCG2 function and provided epithelial cells of the intestinal barrier with ATP to promote UA excretion. Meanwhile, the expression of urate transporters GLUT9 and URAT1 is suppressed by SCFAs [67]. Mannuronate oligosaccharide (MOS) is α-D-mannuronic acid polymer with 1,4-glycosidic linkages. MOS recovered the decrease in beneficial bacteria such as *Lactobacillus* and *Akkermansia* in HUA mice modeled by PO and a high-yeast diet to increase acetates, propionates and isovaleric acids and promoted UA excretion by regulating the protein levels of intestinal GLUT9 and ABCG2 [68]. One novel study employing a compounds–targets–pathways–disease network interpreted that isobavachin could target bile secretion and showed a strong binding affinity for ABCG2 in the treatment of HUA [69]. OCT1 and OCT2 are key transporters in TMAO kinetics, while knockout of these two urate transporters contributed to elevated TMAO levels due to reduced renal excretion [70]. In addition, the elimination of IS and PCS is largely mediated by OAT1/3 in the kidney, as well as ABCG2 in the intestine [71]. On the other hand, dysfunction of these intestinal urate transporters would give rise to HUA. OAT1/3 ablation can lead to substantial alterations in GM composition and can further aggravate renal disease, primarily due to impaired elimination of PCS, along with disturbances in BA metabolism [72]. ABCG2 dysfunction elicited inflammatory responses, resulting in accumulation in intestinal urate and uremic toxins in HUA mice [73]. Thus, intestinal urate transporter activities and GM-derived metabolites reciprocally influence each other.

Collectively, these findings delineate a gut–liver–kidney axis centered on improving GM microecology and positioning GM-derived metabolites as central conduits that link pharmacological interventions to UA metabolism, encompassing hepatic UA biosynthesis, renal and intestinal urate handling, and intestinal barrier integrity. In the treatment of HUA, natural-product-modulated GM exhibited elevated fecal SCFAs concentrations, while concurrently attenuating the generation and accumulation of uremic toxins. Furthermore, SCFA- and BA-mediated restoration of the mucosal barrier restricts the paracellular translocation of nephrotoxic uremic toxins, acting in concert with the coordinated modulation of urate transporter networks, thereby reducing systemic urate burden.

### 4.3. Metabolic Pathways

#### 4.3.1. Amino Acid Metabolism

Microbial colonization may alter the activity of digestive proteases such as trypsin in the intestinal lumen, as well as regulate intestinal barrier function, altogether impacting the uptake and systemic availability of free amino acids in the IT. Also, GM could directly consume host-derived amino acids or secrete essential amino acids fulfilling the host’s nutritional pool [74]. Thus, targeting GM may trigger the intestinal amino acid metabolism, contributing to HUA therapy. During HUA, amino acids are catabolized into key nitrogenous compounds (e.g., UA, urea, and ammonia) that drive systemic nitrogen cycling, while also enhancing renal UA excretion, indicating that they are critical modulators of UA metabolism. For example, polysaccharides in Zhejiang psyllium (AFP) modulated serum endogenous metabolites that are involved in alanine, aspartate, and glutamate metabolism, as well as arginine and proline metabolism, to ameliorate HUA. A high dose of AFP intervention inhibited the rise in levels of L-glutamine, which is a key metabolite connecting alanine, aspartate, and glutamate metabolism to purine metabolism [75]. L-glutamine inhibits XO-dependent UA synthesis and limits intestinal nuclear factor kappa B (NF-κB) activity. Glutamine-derived glutamic acid is attributed to the biosynthesis of proline. In addition, high levels of UA may result in the depletion of circulating proline, while exogenous proline supplementation restrains intestinal redox imbalance and inflammation, and proline-based compounds serve as URAT1 inhibitors [76]. Spearman correlation analysis revealed that the plasma level of L-proline and biosynthesis of branched-chain amino acids (BCAAs) were positively correlated with *Akkermansia*, a core taxa helped to recover kidney function parameters and inflammatory factors following probiotic intervention, which accounted for the relief of nicotinamide adenine dinucleotide phosphate (NADPH)-dependent oxidative stress and cellular apoptosis during HUA. In-depth studies showed that branched-chain α-keto acids are metabolites catabolized from BCAA via transamination and decarboxylation that mediates mammalian target of rapamycin (mTOR) signaling and energy production in kidney fibrosis [77]. Moreover, this finding was in accordance with Liu et al.’s study, suggesting that *Uox*-KO mice experienced a depletion in BCAAs because of an alteration in the abundance of *Akkermansia muciniphila* [78]. It is now clear that when catabolizing excess amino acids, GM taxonomy was inclined to change, promoting the synthesis of secondary BAs, especially DCA, in the liver [79]. Natural phenylethanoid supplementation (tyrosol, hydroxytyrosol, and salidroside) reshaped the GM community, which was correlated with decreased hepatic contents of choline, TMA and TMAO, alongside elevated levels of taurine, a sulfur-containing amino acid involved in BA conjugation. Furthermore, these metabolites were largely implicated in amino acid tRNA biosynthesis and the interconnected metabolic axes of amino acids (glycine/serine/threonine and arginine/proline) in metabolic disorders [80]. In addition, taurine itself exerted an anti-HN effect by improving the microbial-mediated fecal L-Trp level [81]. However, the exact wiring diagram linking specific amino acid disturbances with other GM-derived metabolites remains largely obscure, urging integrative multi-omics and causal inference approaches in the context of HUA progression.

Fangyukangsuan granules, flavonoid extracts of saffron by-products, and coffee leaf extracts displayed regulatory effects on amino acids and their derivatives. Spearman correlation analysis interpreted the association between their differentiated GM pool and amino acid metabolism, and found that natural medicine might influence SCFAs-producing bacteria to take action [17,82]. In addition, the consumption of *Lactobacillus plantarum TY-S8* in HUA mice increased the abundance of *Lactobacillus johnsonii*, which primarily generates indole derivatives, such as indole-3-acetic acid (IAA), indole-3-lactic acid, and indole-3-acetaldehyde [83]. These findings suggest that enlarged populations of beneficial GMs are positively associated with changes in the metabolism of the aromatic amino acid Trp, especially bacteria-derived indoles and their derivatives IAA and IPA. Moreover, this process is potentially mediated by the aryl hydrocarbon receptor in response to natural product interventions. Thereafter, this leads to the suppression of Toll-like receptor 4 (TLR4)/NF-κB/NLRP3 and subsequent pyroptosis and promotion of UA excretion by enhancing intestinal ABCG2 function, thus boosting host immunity [84]. A trial demonstrated that Trp participated in the action of glycine by enhancing the metabolism of glycine to creatinine, ultimately reducing sUA levels and promoting renal UA excretion in mild HUA patients. However, Trp alone did not exert beneficial effects in adjusting UA metabolism, indicating a potential indirect mechanism of Trp in this process [85]. Given that this trial was limited by size, this suggested the necessity to further elucidate the precise molecular mechanisms by which Trp and its metabolites enhance UA excretion. The reduction in opportunistic bacteria *Desulfovibrio* growth showed a strong correlation with Trp metabolism alteration. Moreover, this remodeled GM composition was significantly associated with the attenuation of inflammation involving the TLR4/NF-κB signaling pathway during HUA [86]. Recent studies also have pointed out that natural-product-derived EVs can be absorbed by GM and cross the epithelial barrier, thereby demonstrating potential for maintaining intestinal balance. *Atractylodes macrocephala*-derived EVs restored beneficial microbial communities, thereby modulating Trp metabolism to increase indole derivative production. Consequently, these vesicle-mediated effects reinforced gut barrier function and conferred anti-inflammatory properties by the Th17 cell differentiation signaling cascade [87].

#### 4.3.2. Lipid Metabolism

The lipid profiles turned out to be intimately correlated to the HUA degree, which might have an interplay with the GM and its metabolites. One Chinese large-scale randomized controlled trial demonstrated that GM-produced metabolites are actively involved in lipid metabolism during metabolic dysfunction [88]. BAs promoted intestinal absorption of lipids and their metabolic intermediates (e.g., glycerophospholipids) via FXR signaling [89]. Glycerophospholipid metabolism regulates the synthesis and breakdown of glycerophosphate, and consequently increases fatty acid oxidation and mitochondrial oxidative phosphorylation during HUA. Oral levan intervention upregulated the abundance of beneficial bacteria (e.g., *Muribaculaceae* and *Lactobacillus*) and decreased the proliferation of pathogenic bacteria (e.g., *Escherichia_Shigella* and *Proteus*). This expansion of the SCFA- and BA-producing GM pool demonstrated a strong correlation with eleven serum metabolites involved in glycerophospholipid metabolism, including Pc(p-16:0/18:0), nonaprenyl-4-hydroxybenzoate, PC(36:2), Ps(15:0/24:1(15z)), and Lyso-PC(18:0), in HUA rats [90]. Meanwhile, in order to combat HUA, one peptide, Leu-Gly-Asp-Phe, derived from sunflower capitulum protein hydrolysate, significantly altered the lysophosphatidylcholines level, a key intermediate in glycerophospholipid metabolism, and eliminated aromatic uremic toxins such as p-cresol glucuronide and phenylacetyl-glycine [91]. Sphingolipid metabolism has an interconnection with glycerophospholipid metabolism, especially during sphingomyelin synthesis. The development of HUA led to alterations in genera such as *Bacteroides* and *Lactobacillus*, which in turn resulted in dysregulated sphingolipid metabolism. This dysregulation subsequently contributed to excessive inflammation and reduced CD16^+^ expression on CD14^−^CD16^+^ monocytes [92]. Rare ginsenosides, including Rg3, Rk1, Rg6, and Rg5, exerted anti-HUA effects by remarkably altering levels of ceramide and sphingosine-1-phosphate, thereby restoring sphingolipid metabolism [93].

Fenofibrate, a peroxisome proliferator-activated receptor (PPAR)α agonist anti-hyperlipidemia drug, significantly decreased UA levels and raised urine PH levels [94]. Likewise, theaflavins, as dual agonists of PPARα and PPARγ, displayed a strong effect on XO inhibition and bacterial metabolite-related metabolism via the gut–liver axis [95]. Thus, PPARs are key modulators of lipid disorder in the context of HUA. Upon activation, PPARα and PPARγ translocate from the cytoplasm to the nucleus, where they respectively orchestrate the regulation of genes involved in fatty acid oxidation and lipid transport and disposal. Moreover, SCFAs, especially butyrates, serve as substrates for lipid synthesis by being converted into acetyl-CoA and promote fatty acid oxidation, potentially via activation of AMPK/PGC-1α/PPAR signaling. Peptides derived from *Lonicera japonica* Thunb. activated the PGC-1α/PPARγ/ABCG2 axis, potentially by restoring cecal butyrate contents [96]. Similarly, chicory promoted intestinal UA excretion through the activation of the PPARγ-ABCG2 pathway, mediated by elevated intestinal butyrates [97].

#### 4.3.3. Glucose Metabolism

Emerging mechanistic studies revealed a bidirectional interplay in which elevated UA impairs glucose metabolism through oxidative stress, inflammation, and urate transporters dysregulation, whereas insulin resistance, the key manifestation in glucose imbalance, in turn diminishes renal urate excretion, thereby perpetuating a vicious metabolic cycle between type 2 diabetes and HUA [98]. In addition, lipid metabolism has an intimate relationship with glucose metabolism. High-fat diet (HFD)-induced HUA models have been shown to induce glucose intolerance and reduce insulin resistance, which was associated with insufficient SCFAs generation involving free fatty acid receptor (FFAR) 2/3-related pathways and AMPK-associated responses [99]. Also, He et al. demonstrated that this disrupted glucolipid metabolism which was induced by perturbed intestinal acetates [100]. Moreover, SCFAs activate FFAR 2/3 on colonic L-cells, which promotes the release of glucagon-like peptide-1 (GLP-1), leading to increased insulin secretion and decreased glucagon secretion. GLP-1 agonists act partly by inhibiting renal proximal tubular Na+/H+-exchanger type 3, thereby promoting UA excretion and reducing sUA levels. Polysaccharides from *Cyclocarya paliurus* leaves raise acetate and butyrate levels, leading to increased GLP-1 production and improved insulin sensitivity through regulation of β-cell apoptosis [101].

In HUA, glycolysis dependent on cathepsin B regulates the expression of urate transporters, specifically downregulating URAT1 and GLUT9, and upregulating ABCG2, which in turn leads to improved UA excretion in the kidney [102]. Punicalagin enhanced urate transporter abilities in the intestine and kidney via inhibiting mitogen-activated protein kinase (MAPK)/NF-κB signaling, restricting gut dysbiosis that otherwise triggered LPS accumulation, and normalizing renal metabolites involved in glycolysis and the pentose phosphate pathways, collectively treating HUA. However, the correlation between renal metabolites and GM following punicalagin intervention remained elusive [103]. High levels of fructose are absorbed through the small intestine and finally metabolized in the liver, where they activate glycolysis, and could lead to the progression of HUA associated with chronic inflammatory responses. *Sphacelotheca reiliana* polysaccharides decreased the abundances of *Bacteroidetes* and *Proteobacteria* in the intestine, improved glycolysis/gluconeogenesis metabolic pathways, and corrected l-histidine and hippuric acid contents in high-fructose diet-induced HUA mice [104]. Moreover, sustained urate accumulation would give rise to the production of monosodium urate (MSU) crystals, which are prone to deposit in the joints, bones and kidneys, resulting in gout and renal dysfunction. This is often accompanied by either acute or chronic low-grade inflammation and cumulative redox substances. GLUT1-mediated glycolysis and de novo glucose uptake are essential for the induction of NLRP3 inflammasome activation and IL-1β production elicited by MSU crystals [105]. Overall, these findings suggested that bacterial metabolites associated with glycolysis may be a key target of natural products in the treatment of HUA.

### 4.4. Immunomodulation Regulators

**Figure 3:**
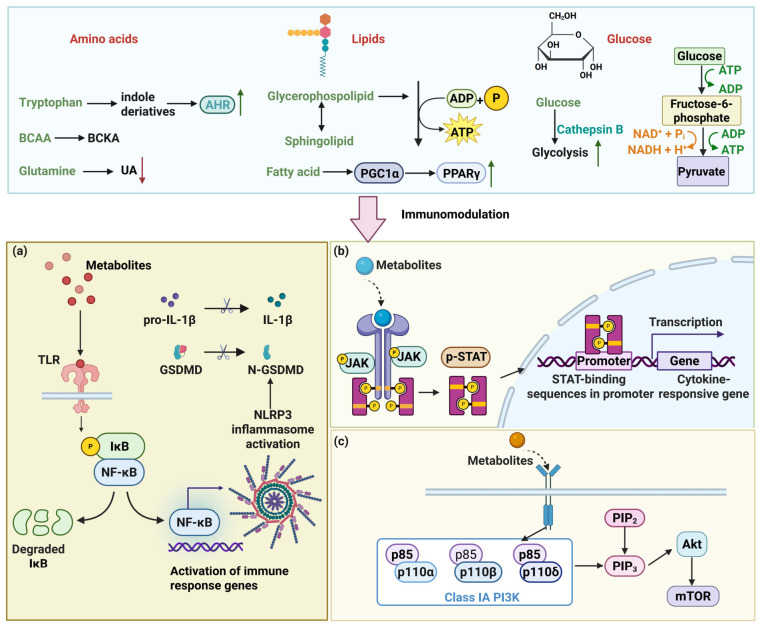
GM-derived metabolites are involved in immunological regulation. Microbial metabolites lead to metabolic changes in amino acids, lipids, and glucose, along with inflammation in the progression of HUA via regulation of Toll-like receptor 4 (TLR4)/ nuclear factor kappa B (NF-κB)/ NOD-like receptor pyrin domain-containing 3 (NLRP3) (**a**), Janus kinase 2 (JAK2)/ signal transducer and activator of transcription 3 (STAT3) (**b**), and Phosphatidylinositol-3-kinase (PI3K)/ protein kinase B (AKT)/ mammalian target of rapamycin (mTOR) (**c**) signaling pathways. ↑, upregulate; ↓, downregulate.

Dynamic interaction between gut metabolites and organs has been shown to significantly influence human health via direct or indirect ways, resulting in alterations in both local and systemic immune responses, as depicted in Figure 3 [106]. The destruction of the intestinal barrier allows lipopolysaccharides (LPSs) in the cell walls of Gram-negative bacteria to enter the circulating bloodstream. Mechanistic studies have demonstrated that patients with impaired intestinal barrier function exhibit elevated levels of bacterial EV-associated LPSs in the circulating blood [107]. Mechanistic studies have demonstrated that damaged intestinal walls release LPS, which acts as a pathogen-associated molecular pattern (PAMP), and the lipid A component of LPS is recognized by LPS-binding protein (LBP). Upon LBP recognization, several LPS components are transferred to CD14, a GPI-anchored protein localized in cholesterol- and sphingolipid-enriched plasma membrane nanodomains. The CD14–LPS complex drives TLR4 activation through recruiting myeloid differentiation protein 2 (MD2), ultimately leading to the formation of the TLR4/MD2/LPS signaling complex. Subsequently, TLRs trigger host defense responses via the Toll/interleukin-1 receptor (TIR) domain, recruiting the adaptor protein myeloid differentiation factor 88 (MyD88) and MAPKs. This signal transduction contributes to the phosphorylation of the inhibitory kappa B kinase (IKK) complex and the activation of NF-κB. After exposure to MSU crystals, which are well known as damage-associated molecular patterns, NLRP3 inflammasomes are driven to be formed and activated, along with the cleavage of pro-IL-1β to active IL-1β and pyroptosis. In particular, NLRP3 interacts with the adaptor protein apoptosis-associated speck-like protein to activate caspase-1 and assemble the NLRP3 inflammasome. Later on, its activation results in the transformation of pro-IL-1β to mature (active) IL-1β production. Thereafter, pro-inflammatory mediators, such as IL-6 and IL-8, are activated and lead to the infiltration of immune cells such as macrophages and neutrophils [108].

Natural-product-derived SCFAs are the most well-studied metabolites influencing UA-associated inflammatory processes. SCFAs maintain both intestinal and systemic immunity via eliminating the generation of reactive oxygen species (ROS) and subsequently suppressing the activation of NF-κB and the NLRP3 inflammasome [109]. Dietary fiber and its metabolite acetate participated in the resolution of inflammation by downregulating NF-κB activity, increasing the production of anti-inflammatory mediators, including IL-10, TGF-β, annexin A1, and enhancing efferocytosis [110]. Moderate intake of crude fiber could upregulate the growth of probiotics *Lactobacillus* and reduce the serum LPS level; however, it had a limited effect on TLR4 and MyD88 mRNA levels against high-protein diet-induced HUA [111]. Hexapeptides from *Apostichopus japonicus* hydrolysate restrained NLRP3 inflammasome activation and pro-inflammatory mediator expressions like IL-1β and TNF-α, while enriching SCFAs-producing taxa including *Eubacterium* and *Lactobacillus*. Notably, these effects were closely linked to the modulation of renal miRNA profiles, suggesting a potential miRNA-mediated mechanism underlying its renoprotective action [112]. Indeed, as the major SCFAs producer, certain probiotic strains belonging to *Lactobacillus* have been linked with the alleviation of inflammatory status via the TLR4/NF-κB/NLRP3/IL-1β pathway in the relief of UA overproduction [113,114]. Alginate promoted the growth of *Pediococcus acidilactici* LW1–1, a probiotic strain with demonstrated XO inhibitory activity and a favorable safety profile. This probiotic, in turn, enriched butyrate-producing taxa, including *Akkermansia*, *Enterocloster*, and *Bacteroidota*. The resulting elevation in butyrate level suppressed renal and intestinal inflammation via inactivation of the NLRP3 inflammasome, while also reprogramming serum metabolites involved in UA biosynthesis and lipid and amino acid metabolism, suggesting a rejuvenation of multi-organ crosstalk by this polysaccharide [115]. In addition, allicin [116], anserine [117] and ginsenoside Rg1 [118] all treated HUA with a strong correlation with NLRP3 inflammasome inactivation.

The binding between GPCRs and SCFAs inhibits cyclic AMP-dependent signaling while simultaneously triggering downstream pathways, including signal transducer and activator of transcription 3 (STAT3) and mTOR signaling, in intestinal epithelial cells. During HUA, inflammatory signals activate Janus kinase (JAK) receptors, and then JAK phosphorylates STAT. Afterwards, p-STAT enters into the nucleus and participates in the regulation of macrophage activities and various inflammatory cofactors. Particularly, STAT3 is recognized as a central player in the macrophage-induced inflammatory process in renal diseases via the JAK/STAT and NF-κB signaling pathways [119]. *Clostridium butyricum* and its metabolite butyrates rescued the depletion of butyrate-producing bacteria and improved host immunity by reducing the number of iNOS^+^ macrophages, while increasing the percentage of CD206^+^ macrophages in the footpads, intestines and spleens of *Uox*-KO mice. Furthermore, this inflammation-relieving reaction was controlled by the inhibition of the miR-146a-mediated JAK2/STAT3 pathway [120]. *Lactiplantibacillus plantarum* MPB-65 combined with epicatechin significantly reversed HUA-induced GM dysbiosis along with SCFAs changes to suppress kidney inflammation through the JAK2/STAT3 signaling pathway [121]. Additionally, a kiwifruit-derived synbiotic with a combination of *Lactiplantibacillus plantarum LP220* and kiwifruit powder produced propionates and butyrates and reduced LPS and IL-1β levels to decrease UA biosynthesis and upregulate urate excretion to treat HUA [122].

Phosphatidylinositol-3-kinase (PI3K)/protein kinase B (AKT) signaling is a classic pathway of promoting the activity of immune cells. PI3Ks are a family of lipid kinases that catalyze the phosphorylation of the 3′-hydroxyl group on the inositol ring of phospholipids, with class I PI3K being the most extensively researched. Class IA PI3K, mediated by its p85 regulatory subunit, is recognized by cell membrane receptors, leading to the subsequent activation of the PI3K p110 catalytic domain by binding to intracellular phosphorylated tyrosine residues of the epidermal growth factor receptor. Once activated, PI3K p110 phosphorylates PIP2 substrate and other proteins to generate the substrate PIP3, which modulates its downstream AKT-mTOR signaling pathway. Flavonoids (luteolin, chrysoeriol, sakuranetin) from the combined polyphenols of medicinal pair Lonicerae flos and Lonicerae Caulis altered intestinal metabolites involved in Trp metabolism and BA secretion and enhanced intestinal immune balance through PI3K-Akt and MAPK signaling during gout [123]. Also, the alterations of urine metabolites by verbenalin were related to amino acid biosynthesis and purine nucleotide metabolism. Furthermore, verbenalin attenuated inflammatory responses by inhibiting PI3K-AKT and MAPK signaling against gout [124]. On the other hand, the increased levels of uremic toxins are inductive of inflammatory responses in kidney damage [125]. TMAO leads to the activation of the PI3K-AKT-mTOR pathway, resulting in inflammation and oxidative stress [126]. Chlorogenic acid increased SCFAs contents and reduced the serum LPS level in PO- and HX-induced HUA mice. In addition, it eliminated TMAO-related bacteria, thereafter inhibiting the PI3K-AKT-mTOR cascade during HN [127].

**Table 1:** Summary of the effects of natural products on gut microbial metabolites in HUA.

Ref.	Natural Products	Experimental Models	Gut Microbiota Changes	Altered Microbial Metabolites	Effects on sUA and Related Outcomes
Yu H et al. [28]	Epigallocatechin gallate	PO-induced HUA mice	↓*Lactobacillus*, ↑*Bifidobacterium*, *Faecalibaculum*	↑PGE2	↓sUA, renal *Oct1*, *Urat1*, *Glut9* ↑renal *Oat1*
Pan L et al. [29]	Berberine	HUA rodents induced in various ways: (1) xanthine and PO, (2) fructose and PO, (3) sodium glutamate, (4) yeast and adenine	↑*Bacteroides* ↓*Coriobacteriaceae_UCG-002*	↑succinic acids, propionates	↓plasma UA, IMP, inosine, HX, xanthine, hepatic AMPD2 ↑plasma AMP
Li YM et al. [36]	Insoluble fiber from barley leaves	Adenine- and PO-induced HN mice	↑*Bacteroides*, *Alloprevotella*, *Eisenbergiella*	↑SCFAs	↓sUA, CRE, oxidative stress
Xu YY et al. [40]	Camellia japonica bee pollen polyphenols	PO-induced HUA mice	↑*Lactobacillus*, *Clostridium*	↑SCFAs	↓sUA, XO, CRE, BUN, oxidative stress
Hung TV et al. [44]	Guar gum, partially hydrolyzed guar gum	Adenine-induced CKD mice	↑*Lactobacillus* spp., *Clostridial cluster* IV, *Bifidobacterium* spp.	↑SCFAs	↓Urea, ammonia
Wu GZ et al. [45]	Coix seed oil	PO- and HX-induced HUA mice	↑*norank_f__Muribaculaceae*, *Akkermansia*, *Lachnospiraceae_NK4A136_group*, *Prevotellaceae_UCG-001* ↓*Lactobacillus*, *Bacteroides*, *Dubosiella*	↑SCFAs	↓sUA, ADA, XO, renal URAT1, GLUT9 ↑renal OAT1, ABCG2
Zhan-g TT et al. [46]	Oleanolic acid	PO- and HX-induced HUA mice, FMT	↑*Rikenellaceae_RC9_gut_group*, *Turicibacter*, *Akkermansia*, *Allobaculum*	↑acetates, butyrates	↓sUA, XO, CRE, renal URAT1, GLUT9 ↑renal ABCG2
Wang ST et al. [53]	Secoisolariciresinol diglucoside from flaxseed	PO- and HX-induced HUA mice	↓*Desulfovibro* ↑ *Ruminococcus*, *Prevotellaceae_UCG-001*	↑propionates, butyrates ↓CA, DCA, GUDCA	↓sUA, XO, renal URAT1, GLUT9 ↑renal OAT1, ABCG2
Liu CQ et al. [58]	Theabrownin	Phenanthrene-induced HUA mice	↓*Clostridium_XIVb*, *Bacteroides*, *Roseburia* ↑*Prevotella*, *Saccharibacteria*	↓TCA, GCA, CDCA, DCA, TDCA, TUDCA	↓sUA
Guo YJ et al. [62]	Inulin	*Uox*-KO HUA mice	↑*Akkermansia*, *Ruminococcus*, *Parasutterella*, *Bifidobacterium*	↑SCFAs ↓IS, PCS	↓sUA, XO
Xia JA et al. [63]	Oat-resistant starch	Adenine-induced CKD mice with FMT from CKD patients	↑*Lactobacillus*, *norank_f_Muribaculaceae*, *Romboutsia* ↓*Faecalibaculum*, *Bifidobacterium*, *Aerococcus*	↑SCFAs ↓IS, PCS, indole, p-cresol	↓sUA, CRE, urea nitrogen
Peng BZ et al. [64]	Quercetin	*Uox*-KO HUA mice	↓*Blautia*, *Lachnospiraceae*	↓uremic toxins (e.g., 3-phenyllactic acid, hippuric acid, N-acetyl-l-phenylalanine)	↓sUA, CRE, BUN
Xie J et al. [65]	Panax notoginseng saponins	Adenine-induced CKD rats	↑*Bacteroides*, *Halomonas*, *Lactobacillus*, *Butyricimonas*, *Faecalibacterium* ↓*Ruminococcaceae*, *Escherichia-Shigella*, *Bacteroidaceae*	↓TMAO	↓CRE, Urea, urine albumine ↑GFR
Wei BQ et al. [68]	Mannuronate oligosaccharide	PO- and high-yeast diet-induced HUA mice	↓*Tyzzerella*, *Bilophila* ↑*Muribaculum*, *Ruminococcus*, *Faecalibaculum*, *Clostridia_UCG−014*	↑acetates, propionates, isovaleric acids	↓sUA, renal URAT1, GLUT9
Wu D et al. [75]	Zhejiang psyllium polysaccharides	PO-, HX- and adenine- induced HUA rats	↑*Lachnospiraceae*, *Oscillospiraceae*, *Limosilactobacillus*, *Ligilactobacillus*, *Eubacterium*_sp., *Bacilli*	↓L-glutamine, L-arginine ↑LysoPC(18:1), LysoPC(20:4), LysoPE(18:0)	↓sUA, CRE, BUN
Zhan XJ et al. [80]	Tyrosol, hydroxytyrosol, salidroside	High-fructose diet- induced mice with metabolic syndrome	↓*Proteobacteria* ↑*Actinobacteria*	↓choline, TMA, TMAO ↑taurine	↓sUA, XO
Yang XJ et al. [81]	Taurine	adenine and ethambutol hydrochloride-induced HN rats	↑*Lactobacillus*, *Lachnospiraceae_NK4A136_group*	↓L-Trp	↓sUA, CRE, urea, renal URAT1, GLUT9 ↑renal OAT1, ABCG2
Xu M et al. [90]	Levan	PO- and HX-induced HUA rats	↑*Muribaculaceae*, *Faecalibaculum*, *Roseburia*, *Lactobacillus*	↑Pc(p-16:0/18:0), PC(36:2), Ps (40:0), Ps(15:0/24:1(15z)), Ps(15:0/22:1(13z)) ↓Lyso-PC(18:0) LysoPC(20:3(8z,11z,14z)/0:0),	↓sUA, XO, ADA, CRE, BUN ↑renal OAT1, ABCG2
Ji XY et al. [93]	Rare ginsenosides	PO-induced HUA mice	↑*Lactobacillus*	↓ceramide ↑sphingosine-1-phosphate	↓sUA, XO, CRE, BUN
Yang HX et al. [96]	Peptides derived from *Lonicera* *japonica* Thunb.	PO- and HX-induced HUA mice	↑*Clostridia*, *Prevotella*, *Lachnospiraceae_NK4A136_group*	↑SCFAs	↓sUA, XO, CRE, BUN
Zhou XF et al. [127]	Chlorogenic acid	PO- and HX-induced HUA mice	↑*Bacteroides*, *Alistipes*, *Butyricimonas* ↓*Muribaculum*, *Faecalibaculum*, *Aeromonas*	↑SCFAs ↓LPS	↓sUA, XO, CRE, BUN ↑renal OAT1, ABCG2

BUN, blood urea nitrogen; CRE, creatinine; HX, hypoxanthine; PO, potassium oxonate; sUA, serum uric acid; XO, xanthine oxidase; ↓, downregulate; ↑, upregulate.

## 5. Clinical Investigation for Natural Products Targeting Gut Microbiota and Its Derived Metabolites

In accordance with experimental evidence, HUA perturbs GM and its metabolite profiles. Analysis of 16S rRNA sequencing data across multiple Chinese cohorts revealed that individuals with HUA and gout harbor GM signatures distinct from those of healthy controls. These patients exhibited an enrichment of pro-inflammatory taxa, such as *Fusobacterium* and *Bilophila*, along with a concomitant depletion of anti-inflammatory and metabolically beneficial bacteria, including the *Christensenellaceae R-7* group, *Anaerostipes*, and *Collinsella* [128]. Moreover, during the progression from asymptomatic HUA to gout, elevated UA may alter the production of SCFAs patterns due to dynamic changes in GM composition [129]. Although both patient groups demonstrated a shared heightened abundance of *Prevotella*, asymptomatic HUA patients tended to harbor more SCFAs-producing bacteria, especially those responsible for butyrate and propionate production [130]. In contrast, fecal metagenome profiling of gout patients not only showed a decline in butyrates-producing species but also revealed a lowered abundance of genes involved in propionate and butyrate biosynthesis [131]. These data indicated a relatively balanced enterotype colonized in asymptomatic HUA patients rather than gout patients.

Clinical evaluation of uremic toxin levels is essential for CKD diagnosis. Serum TMAO concentrations exhibited a strong negative correlation with the estimated glomerular filtration rate (eGFR), while showing a significant positive correlation with markers of renal impairment, including sUA, urea, creatinine (CRE), and BUN [132]. Meanwhile, a marked decrease in urinary TMAO levels was observed, which was correlated with *Bifidobacterium* and *Lactobacillus* [133]. Furthermore, in the fecal samples from CKD patients, PBUTs are associated with lower abundance of SCFAs-generating bacteria, such as *Bifidobacterium* spp. and *Streptococcus* spp., and a higher abundance of bacteria that impairs renal function, including *Enterobacteriaceae* and *Escherichia coli* [134].

Mounting translational evidence has indicated that natural products hold promise for HUA therapy by targeting GM in clinics. In line with preclinical data, supplementation of dietary fiber, with its excellent ability over UA elimination and GM dysbiosis regulation, served as a promising candidate to downregulate PCS and IS levels in CKD patients [135]. CKD patients given a high fermentable fiber diet showed a significant decrease in serum p-cresol levels, while the IS level was not markedly altered [136]. An 8-week, three-arm randomized controlled trial further indicated that an oatmeal-based fiber diet (OM) showed superiority over a resistant starch-based fiber diet (RS) in lowering sUA. Mechanistically, this outcome may be attributed to a significant enrichment of *Dialister* and a concomitant elevation in serum levels of *threo*-syringoylglycerol, a phenolic compound with anti-inflammatory and antioxidant properties [137]. Low-protein diet (LPD) downregulated amino acid catabolism, thereby reducing sUA content and enhancing protein utilization [138]. In addition, according to a longitudinal, prospective, controlled, and interventional study, a combined treatment of LPD and inulin supplementation increased the abundance of Bifidobacteriaceae and decreased the abundance of Enterobacteriaceae at the family level. Additionally, individuals with CKD on trial for 6 months experienced a marked reduction in UA and C-reactive protein levels in serum, circulating TNF-α and NOX2 level in plasma, along with a marked increase in serum bicarbonate [139]. Shotgun metagenome sequencing of GM and targeted fecal metabolome of BAs among middle-aged and elderly Chinese HUA participants in a longitudinal study indicated that dietary lignan intervention helped to decrease *Fusobacterium mortiferum*, *Blautia* sp. *CAG-257*, and downstream BA products (e.g., NorCA, GCDCA, and GUDCA) [140]. Dietary fat diacylglycerol supplementation for two months decreased sUA in HUA male athletes. A lipidomics study indicated that this intervention significantly decreased circulating triacylglycerol levels and upregulated serum plasmalogen lipids, which were negatively associated with accumulation of p-cresol [141]. Mild CKD patients taking 6 months of curcumin tend to reduce the accumulation of IS and PCS, interfere with the growth of pathogenic *Escherichia–Shigella*, and mitigate inflammatory factors such as MCP-1 and IL-4 [142]. In a randomized, double-blind crossover trial, six-week synbiotic supplementation led to a statistically significant reduction in the circulating PCS level and a moderate decrease in the IS level, as well as increased fecal beneficial genera *Bifidobacterium* and *Blautia* in 37 patients with stage 4 or 5 CKD [143]. In addition, probiotic supplementation may lower PCS levels, thereby protecting the intestinal epithelial barrier in patients with CKD [144]. Based on a two-month randomized, double-blind, placebo-controlled trial, co-administering Probio-X with febuxostat not only aided to enlarge the SCFAs-producing GM population, but also facilitated the growth of health-promoting microbes, such as *Bifidobacterium adolescentis*, *Lachnospira eligens* and *Bariatricus comes*, resulting in an enhanced efficacy of febuxostat. This combination could alter metabolic pathways, including BA biosynthesis, porphyrin metabolism, purine metabolism, and riboflavin metabolism, as well as nicotinate and nicotinamide metabolism, to drastically decrease IL-1β and XO levels in gout patients [145]. FMT from healthy mice or donors contributed to a normalized gut community against UA overload [146]. Washed microbiota transplantation (WMT) is refined based on FMT that allows an automatic washing process and provides a safer and more convenient way of bacteria delivery, which was initially standardized in 2019 [147]. A pilot study revealed that the level of harmful intestinal metabolite LPS was downregulated utilizing WMT to alleviate gout symptoms [148]. Accordingly, Table 2 summarizes current therapeutic approaches for HUA and related comorbidities that modulate GM and its metabolites, including natural-product-based interventions.

Overall, treatments including synthetic drugs, natural products and probiotics/synbiotics efficiently rescue kidney injury by enhancing beneficial microbes and eliminating pathogenic bacteria and their derived metabolites. However, these clinical trials are limited in size, and their mechanisms of action are not fully elucidated. Moreover, although these interventions are inductive to GM and metabolites changes, there is a lack of standards for evaluating these outcomes in these trials. With respect to the chronic metabolic state, in humans, which differed from animal models, large-scale, multicenter, randomized clinical trials are warranted to assess the efficacy of potential anti-HUA drugs in the real world with standardized GM outcome measures.

## 6. Future Directions for Clinical Translations

While the role of GM-derived metabolites in HUA has been increasingly characterized, clinical translation remains constrained by the lack of validated, noninvasive biomarkers for reliably identifying at-risk individuals and monitoring treatment responses. Addressing this gap will likely require precision stratification strategies grounded in microbiome science and systems biology. One potential approach involves the combined profiling of GM communities through 16S rRNA gene sequencing or shotgun metagenomics, and the measurement of GM-derived metabolites in serum, feces, or urine, such as SCFAs, uremic toxins, and LPSs, via untargeted or targeted metabolomics. These integrated data can help delineate specific dysbiotic signatures associated with HUA and renal injury, while capturing functional host–microbiome interactions that are frequently disrupted in these conditions [157]. Furthermore, merging multiple omics layers could yield composite indices that reflect dysfunction of the gut–kidney axis comprehensively [158]. Due to the high reliance of microbiome analysis on algorithms and computational tools, it is essential to establish standards for the use and operation of these tools. Appropriate benchmarking, open-source availability, a straightforward installation process, and a clear user interface all contribute to enhancing the reproducibility and interpretability of results. Moreover, with the integration of modern metatranscriptomic and metaproteomic technologies, it helps validate sequencing data reliability and enables more comprehensive functional profiles of environmental samples [159].

Translating GM-oriented strategies into clinical practice for HUA necessitates a coordinated and standardized roadmap. First, the validation of mechanistic insights in preclinical models constitutes an important foundation. This process can be strengthened by applying multi-omics technologies, such as 16s rRNA sequencing, metabolomics, and transcriptomics, alongside functional assays, to ensure relevance to human disease. Notably, at this phase, it is important for researchers to identify and confirm the disease GM biomarkers. Carr et al. introduced microbial community-scale metabolic models (MCMMs) that incorporate cooperative trade-off FBA (ctFBA), a mechanistic framework for pinpointing specific GM metabolites that play a causal role in disease progression, thereby defining these metabolites as HUA-specific therapeutic targets [160].

Second, early phase clinical trials should be pursued to evaluate the feasibility of GM-targeted interventions, while employing changes in sUA levels and relevant microbial metabolites profiles as biomarker-based surrogate endpoints. In addition, these trials are encouraged to emphasize the integration of gut microbiome enterotypes into patient selection criteria, thereby enabling metabolite-specific therapies [161]. These studies may help with the design of larger-scale trials by refining patient selection, particularly enabling the enrollment of individuals with early, asymptomatic HUA for timely microbiota-tailored intervention.

Third, ensuring the long-term safety of GM-targeting interventions constitutes a critical and unresolved priority. Although retrospective cohort studies have confirmed that long-term FMT is generally well-tolerated, equivalent safety data for probiotics and natural products remain scarce, leaving substantial uncertainty [162]. To bridge this evidence gap, the International Scientific Association for Probiotics and Prebiotics recommends that the intervention’s quality (purity, potency, and identity) should be first ensured through third-party verifications and transparently communicated on product labels. In parallel, all clinical trials should be required to incorporate scientifically rigorous long-term safety endpoints and to collect and report adverse events. A sentinel safety biomarker panel is recommended that integrates gut barrier integrity markers (e.g., ZO-1, Claudin-1, Occludin), microbial drivers of systemic immune activation, and metagenomic and metatranscriptomic surveillance of resistome expansion and horizontal gene transfer events besides conventional organ function tests (BUN, CRE, ALT, AST, etc.). Furthermore, dedicated research is urgently needed to delineate high-risk subpopulations, such as middle-aged and elderly males and patients at specific physiological stages or with comorbid conditions who require closer long-term monitoring and follow-up, thereby enabling a risk-stratified safety monitoring framework [163].

Moreover, the establishment of science-anchored regulatory and policy architectures that can reconcile innovation with safety is essential for accelerating the responsible clinical translation of GM-tailored therapies. This demands a concerted transdisciplinary effort that integrates the expertise of nephrologists, microbiologists, immunologists, nutritional scientists, and computational biologists to address HUA from a holistic perspective.

Overall, personalized GM-targeted interventions leveraging natural products as safe and sustainable therapeutic strategies hold particular promise, provided that the above roadmap—encompassing multi-omics stratification, rigorous safety surveillance, and regulatory harmonization—is diligently pursued. However, the challenges on the road to translation must also be acknowledged. One is that natural products are biotransformed by the GM into bioactive metabolites [164]. Given that GM colonization is shaped by age, diet, genetics, and health status, substantial interindividual variability exists in the microbial response to natural products and their resultant therapeutic effects. Another is the inherent complexity of natural products. Taking inulin as an example, while it exerts health-promoting effects via SCFAs generation, it can paradoxically exacerbate certain diseases, including colitis, by augmenting pro-inflammatory bacterial components [165]. Furthermore, the therapeutic efficacy of inulin exhibits diurnal variation: evening administration showed superiority over morning intake due to a more pronounced capacity in alleviating the inflammatory responses and improving amino acid metabolism [166]. Despite the poor oral bioavailability of many natural products—as exemplified by quercetin, for which only approximately 3–17% of an orally administered dose is absorbed in humans [167]—their obstacles to clinical translation are rooted in regulatory limitations. Specifically, such compounds are typically marketed as dietary supplement ingredients rather than as FDA- or NMPA-approved drugs, which raises significant concerns regarding product consistency, safety, and long-term effects [168].

## 7. Discussion

The fact is that there is a bidirectional relationship between microbial metabolites and HUA progression. On the one hand, elevated sUA levels can provoke gut dysbiosis and impair intestinal barrier integrity, consequently leading to an altered profile of microbial metabolites. On the other hand, which constitutes the focus of this review, these microbial metabolites may in turn modulate HUA progression through multiple interconnected pathways. It is now acknowledged that SCFAs exert a beneficial role in attenuating HUA, whereas uremic toxins compromise renal function. However, research investigating the causal role of BAs in HUA remains relatively scarce, as their involvement has been primarily characterized in metabolic dysfunction-associated fatty liver disease, a common comorbidity of HUA [169]. Butyrate, serving as a primary energy substrate for colonocytes, is among the most extensively studied SCFAs and has been implicated in the alleviation of HUA. Mechanistic evidence indicates that butyrate can lower sUA levels through a coordinated combination of suppressing UA biosynthesis, promoting urate excretion, and regulating urate transporter activity [170]. Although the concept of the “butyrate paradox” has been recognized in intestinal diseases, studies elucidating the phenomenon in HUA remain notably sparse [171,172]. Yet, for certain metabolites, it remains challenging to ascertain whether they are exclusively microbiota-derived or from other origins such as interventions or the host itself [173].

In addition, the integration of 16S rRNA sequencing with metabolomics is progressively unraveling the mechanistic underpinnings of natural products in the treatment of HUA. However, the majority of studies remain confined to establishing correlations between the sUA-lowering capacity of natural products and concurrent alterations in GM composition and its derived metabolites; consequently, the causal contribution of gut microorganisms to disease amelioration by these natural products remains insufficiently elucidated. Therefore, the application of FMT and antibiotic-induced GM depletion represents a promising strategy to address this limitation. Given the complexity and diversity of GM and their interactions within the host, researchers need to extend beyond individual strains to encompass the relationships and interspecies dynamics among gut microbes.

Although there is substantial preclinical evidence supporting the anti-HUA potential of natural products, to date, only a few agents, such as curcumin, dietary fiber and BBR, have been clinically validated. Moreover, interventions with dietary fibers are garnering increasing global attention for the treatment of HUA and its associated comorbidities through the modulation of the GM and its metabolites, as reflected by the rapidly growing number of registered clinical trials in this area. Despite pharmacokinetic limitations, long-term safety uncertainties, and other challenges associated with natural products discussed in Section 6, the use of comparable doses—specifically, human-equivalent doses aligned between preclinical and clinical settings—is essential to achieving optimal therapeutic efficacy. Moreover, the inherent complexity of metabolic disease pathogenesis challenges the validity of animal models. Approaches that rely solely on single inducers, such as PO, HN, or adenine, cannot comprehensively recapitulate the progressive and multifactorial nature of human disease progression, thereby substantially limiting their translational value for clinical validation.

## 8. Conclusions

Modulating GM-derived metabolites offers a potential strategy for treating HUA and related systemic metabolic disorders. Natural products can regulate the GM community, thereby exerting therapeutic effects against HUA. During the disease process, these agents restore intestinal microbial homeostasis by enriching SCFAs-producing commensals while reducing pathogenic taxa and ameliorating intestinal histopathological alterations, partly through reinforcing tight junction integrity. Moreover, by modulating the GM, these agents reshape the microbial signatures, thereby attenuating metabolite-driven inflammation, potentially through modulation of the TLR4/MyD88, JAK/STAT, and PI3K/AKT/mTOR signaling cascades, and reprogramming systemic metabolite-associated pathways, encompassing amino acid, lipid, and glucose metabolism. In addition, clinical evidence demonstrated that certain natural products hold therapeutic promise in reducing sUA levels and favorably modulating GM and derived metabolite compositions.

Therefore, this study highlights the potential of natural products as a complementary therapeutic strategy for HUA, and provides direction for future studies to advance this valuable area.

## Figures and Tables

**Table 2:** Clinical trials of treatments targeting the gut microbiota against UA overload.

Ref.	Trial Design	At Risk	UA-Lowering Therapy	Daily Dosage	GM Changes	GM-Derived Metabolites Changes	Clinical Parameters	Metabolic Pathway
Lin S et al. [8]	Observational case- controlled	Gout (*n* = 76)	3 months of febuxostat	/	↑*Cytophaga*, *Dorea*, *Clostridium*, *Fecalibacterium*	/	↓UA	↑purine, carbohydrate metabolism
Pan L et al. [29]	Longitudinal prospective interventional	HUA (*n* = 8)	6 months of BBR	0.5 g, bid	↑*Bacteroides* ↓*Clostridium sensu stricto_1*	/	↓sUA, CRE	/
Li TZ et al. [137]	Three-armed, randomized controlled, triple-blinded	HUA (*n* = 95)	8 weeks of oatmeal rice (OM) or resistant starch rice (RS)	Approximately 25 g dietary fiber per day	↑*Dialister* (OM) No statistically significant difference in the abundance of this genus was observed between the RS group and the control group.	↑*threo*-syringoylglyce-rol (both)	Both diets reduced sUA; however, OM showed a greater effect.	/
Lai S et al. [139]	Longitudinal prospective placebo-controlled interventional	CKD (stage 3G–4G) (*n* = 16)	6 months of LPD & inulin	LPD: 0.6 g/kg insulin: 19 g	↑Bifidobacteriaceae ↓Enterobacteriaceae	/	↓sUA, NOX2, CRP, TNF-α ↑bicarbonate	/
Zhuo LB et al. [140]	Longitudinal prospective	HUA (*n* = 2552)	10.5 years (medium) of dietary legnans	/	↓*Fusobacterium mortiferum*, *Blautia* sp. *CAG-257* ↑*Akkermansia muciniphila*	↓GCDCA, GUDCA, TCDCA	↓sUA	/
Zhang F et al. [141]	Longitudinal prospective interventional	HUA (*n* = 33)	2 months of diacylglycerol from soybean	/	/	↓p-Cresol ↑phosphatidyl- choline	↓sUA	↑phospholipid metabolism
Pivari F et al. [142]	Longitudinal prospective interventional	CKD (stage 3a–4) (*n* = 24)	3 or 6 months of curcumin tablet	500 mg/tablet twice a day	↓*Enterobacter* *Escherichia-Shigella*, ↑*Lachnoclostridium*	↓IS, PCS (with no statistical difference)	↓MCP-1, IL-4, IFN-γ	/
Rossi M et al. [143]	Randomized double-blinded placebo-controlled crossover	CKD (stage 4–5) (*n* = 31)	6 weeks of synbiotics	4.5 × 10^10^ CFU 9 × 10^10^ CFU	↑*Bifidobacterium* spp., *Faecalibacterium* spp.	↓PCS, IS	No marked changes in biomarkers of inflammation, oxidative stress	/
Zhao F et al. [145]	Randomized double-blinded placebo-controlled	Gout (*n* = 160)	2 months of Probio-X & febuxostat	3 × 10^10^ CFU	↑*Lachnospira eligens*, *Bariatricus comes* *Bifidobacterium adolescentis*	↓GDCA, GUDCA, GCA	↓sUA, CRE, TG	↓BAs synthesis ↑purine, riboflavin, nicotinate, nicotinmide metabolism
Xie WR et al. [148]	Pilot study	Gout (*n* = 11)	3, 6, 9 times of WMT	200 mL FMT suspension each time	/	↓LPS	↓sUA	/
Deng X et al. [149]	Randomized open-label double-armed	T2DM & CVD (*n* = 76)	12 weeks of empagliflozin	10 mg	↑*Lachnospiraceae*, ↓*Escherichia-Shigella*	↓amino acids phosphosphingolipids, GCDCA	↓UA, IL-6 ↑hematocrit, adipokine	/
Kavyani M et al. [150]	Parallel-group randomized Double-blinded placebo-controlled	MAFLD (*n* = 44)	12 weeks of camelina oil, resistant dextrin	20 g camelina oil, 10 g resistant dextrin	/	↓LPS	↓insulin, HOMA-IR, hs-CRP, MDA, UA	/
Lao BN et al. [151]	Prospective controlled	Obese & CKD stage 3–4 (*n* = 28)	12 weeks of time- restricted LPD	/	↑*Lachnospiraceae*, *Clostridia*, *Verrucomicrobia*, *Akkermansia*, *Oscillibacter*, *Ruminococcaceae*, *Anaerotruncus*	/	↓UA, CysC, TNF-α ↑eGFR, ALB	/
Lin JH et al. [152]	Placebo-controlled double-blinded randomized	HUA & MAFLD (*n* = 82)	2 months of *Lactobacillus fermentum* TSF331, *L. reteri* TSR332, *L. plantarum* TSP05	6.7 × 10^9^ CFU	↑*Lactobacillus*, *Faecalibacterium* ↓*Mogibacterium*, *Catonella*	/	↓sUA, glucose, lipid	/
Ding D et al. [153]	Non-blinded, one-armed intervention prospective	T2DM (*n* = 17)	2 days of FMT from healthy donors	/	↑*Anaerotruncus*	/	↓sUA, HbA1c, glucose ↑postprandial C-peptide	/
Cao C et al. [154]	Observational	UA stones (*n* = 12)	3 months of potassium sodium hydrogen citrate	10 g	↓Fusobacterium ↑Lachnoclostridium, Parasutterella	↑butyrates	↓sUA	↑fatty acid biosynthesis, amino acid metabolism
Kond-ratiuk VE et al. [155]	Randomized controlled	primary gout (*n* = 68)	3 months of Alo & synbiotics	Alo: l300 mg symbiotics: 2.5 × 10^9^ CFU	↑*Lactobacillus* spp., *Pseudomonas* spp.	/	↓sUA, CRP, IL-1β, IL-6, IL-8, TNF-α	/
Kalidin-di RK et al. [156]	Comparative phase IV randomized open-label controlled parallel	CKD (stage 3–4) (*n* = 60)	6 months of Lobun Forte or Renadyl	4.5 × 10^10^ CFU twice a day	/	↓IS (both drugs), ↓PCS (Renadyl)	↓BUN, CRE, GSH, NO, eGFR (both drugs) ↓hsCRP (Lobun Forte)	/

Alo, allopurinol; CFU, colony-forming unit; CRP, C-reactive protein; eGFR, estimated glomerular filtration rate; IFN-γ, interferon-gamma; MCP-1, monocyte chemoattractant protein-1; sUA, serum uric acid; ↓, downregulate; ↑, upregulate.

## Data Availability

No new data were created or analyzed in this study. Data sharing is not applicable to this article.

## Abbreviations

The following abbreviations are used in this manuscript.

ABCG2	ATP-binding cassette transporter G2
ADA	Adenosine deaminase
BA	Bile acid
EV	Extracellular vesicle
FMT	Fecal microbiota transplant
FXR	Farnesoid X receptor
GLUT9	Glucose transporter 9
GM	Gut microbiota
HUA	Hyperuricemia
IS	Indoxyl sulfate
LPS	Lipopolysaccharide
MSU	Monosodium urate
MyD88	Myeloid differentiation factor 88
PCS	P-cresol sulfate
PO	Potassium oxonate
SCFA	Short-chain fatty acid
TJ	Tight junction
TLR4	Toll-like receptor 4
TMAO	Trimethylamine-N-oxide
Trp	Tryptophan
UA	Uric acid
Uox	Urate oxidase
URAT1	Urate transporter 1
XO	Xanthine oxidase

## References

[1] Ahn J.K. (2023). Epidemiology and treatment-related concerns of gout and hyperuricemia in Korean. J. Rheum. Dis..

[2] Emery H.L., Kerby R.L., Rey F.E. (2025). The Central Role of Gut Microbes in Host Purine Homeostasis. Annu. Rev. Microbiol..

[3] Fang Y., Qin M., Zheng Q., Wang K., Han X., Yang Q., Sang X., Cao G. (2024). Role of Bile Acid Receptors in the Development and Function of Diabetic Nephropathy. Kidney Int. Rep..

[4] Agus A., Clément K., Sokol H. (2021). Gut microbiota-derived metabolites as central regulators in metabolic disorders. Gut.

[5] Monnerie S., Comte B., Ziegler D., Morais J.A., Pujos-Guillot E., Gaudreau P. (2020). Metabolomic and Lipidomic Signatures of Metabolic Syndrome and its Physiological Components in Adults: A Systematic Review. Sci. Rep..

[6] Wang Y., Huang B., Wei X., Guan Y., Li L., Zheng Y., Sun W. (2026). Gut microbiota metabolic reprogramming drives the development of metabolic diseases in the host. Gut Microbes.

[7] Yu Y., Liu Q., Li H., Wen C., He Z. (2018). Alterations of the Gut Microbiome Associated With the Treatment of Hyperuricaemia in Male Rats. Front. Microbiol..

[8] Lin S., Zhang T., Zhu L., Pang K., Lu S., Liao X., Ying S., Zhu L., Xu X., Wu J. (2021). Characteristic dysbiosis in gout and the impact of a uric acid-lowering treatment, febuxostat on the gut microbiota. J. Genet. Genom..

[9] Wang Z., Wang S., Chen Y., Liang L., Yang L., Zeng L. (2025). Ultrasound-assisted betaine-based natural deep eutectic solvents for green extraction of total phenols and flavonoids from *Lithocarpus litseifolius*: Mechanistic insights and anti-hyperuricemic applications. Food Res. Int..

[10] Tawfick M.M., Xie H., Zhao C., Shao P., Farag M.A. (2022). Inulin fructans in diet: Role in gut homeostasis, immunity, health outcomes and potential therapeutics. Int. J. Biol. Macromol..

[11] Liu X., Tong X., Zou Y., Lin X., Zhao H., Tian L., Jie Z., Wang Q., Zhang Z., Lu H. (2022). Mendelian randomization analyses support causal relationships between blood metabolites and the gut microbiome. Nat. Genet..

[12] Liu Z.Q., Sun X., Liu Z.B., Zhang T., Zhang L.L., Wu C.J. (2022). Phytochemicals in traditional Chinese medicine can treat gout by regulating intestinal flora through inactivating NLRP3 and inhibiting XOD activity. J. Pharm. Pharmacol..

[13] Meng W., Dai H., Li R., Zhang J., Lin Y., Dong P. (2026). Tissue-specific metabolism of Naringin and its modulation of the UA metabolism and gut microbiota in hyperuricemia mice. Bioorg Chem..

[14] Zhang K., Sun S., Qiu H., Zhao H., Zhao H., Shen Y., Wang Y., Zhang Y. (2026). Viscum coloratum polysaccharide ameliorates hyperuricemic nephropathy by upregulating Nrf2 to inhibit TGF-β1/Smad3 and NLRP3/ASC/Caspase-1 pathways. J. Ethnopharmacol..

[15] Wu D., Chen R., Zhang W., Lai X., Sun L., Li Q., Zhang Z., Cao J., Wen S., Lai Z. (2022). Tea and its components reduce the production of uric acid by inhibiting xanthine oxidase. Food Nutr. Res..

[16] Li J., Li D., Chen Y., Chen W., Xu J., Gao L. (2023). Gut Microbiota and Aging: Traditional Chinese Medicine and Modern Medicine. Clin. Interv. Aging.

[17] Li H., Shen N., Ren J., Yang S., Chen Y., Gao Z. (2024). Biotransformation characteristics of urate-lowering probiotic fermented apple juice and potential regulatory mechanisms for ameliorating hyperuricemia via mediating gut microbiota and metabolic pathways. Food Chem..

[18] Kikusato M., Xue G., Pastor A., Niewold T.A., Toyomizu M. (2021). Effects of plant-derived isoquinoline alkaloids on growth performance and intestinal function of broiler chickens under heat stress. Poult. Sci..

[19] Qi X., Ma Y., Guan K., Zhao L., Ma Y., Wang R. (2024). Whey Protein Peptide Pro-Glu-Trp Ameliorates Hyperuricemia by Enhancing Intestinal Uric Acid Excretion, Modulating the Gut Microbiota, and Protecting the Intestinal Barrier in Rats. J. Agric. Food Chem..

[20] Wu C., Hu Q., Peng X., Luo J., Zhang G. (2023). Marine Fish Protein Peptide Regulating Potassium Oxonate-Induced Intestinal Dysfunction in Hyperuricemia Rats Helps Alleviate Kidney Inflammation. J. Agric. Food Chem..

[21] Han R., Li Y., Guo Y., Ren M., Shan M., Mao T., Qi X., Li Y., Tian Z., Fu T. (2025). Alginate ameliorates hyperuricemia in mice by restoring hyperuricemia-induced renal and intestinal dysfunctions. Int. J. Biol. Macromol..

[22] Song M., Chen L., Dong C., Tang M., Wei Y., Lv D., Li Q., Chen Z. (2025). Alginate Oligosaccharide and Gut Microbiota: Exploring the Key to Health. Nutrients.

[23] Wu J., Wei Z., Cheng P., Qian C., Xu F., Yang Y., Wang A., Chen W., Sun Z., Lu Y. (2020). Rhein modulates host purine metabolism in intestine through gut microbiota and ameliorates experimental colitis. Theranostics.

[24] Grelska A., Sharan D., Light S.H. (2023). Purine-ifying uric acid by gut microbes. Cell Chem. Biol..

[25] Liu Y., Zhou Z., Jarman J.B., Chen H., Miranda-Velez M., Terkeltaub R., Dodd D. (2025). Gut bacteria degrade purines via the 2,8-dioxopurine pathway. Nat. Microbiol..

[26] Fu Y., Luo X.D., Li J.Z., Mo Q.Y., Wang X., Zhao Y., Zhang Y.M., Luo H.T., Xia D.Y., Ma W.Q. (2024). Host-derived *Lactobacillus plantarum* alleviates hyperuricemia by improving gut microbial community and hydrolase-mediated degradation of purine nucleosides. eLife.

[27] Zhou Y., Zeng Y., Wang R., Pang J., Wang X., Pan Z., Jin Y., Chen Y., Yang Y., Ling W. (2024). Resveratrol Improves Hyperuricemia and Ameliorates Renal Injury by Modulating the Gut Microbiota. Nutrients.

[28] Yu H., Lou Z., Wu T., Wan X., Huang H., Wu Y., Li B., Tu Y., He P., Liu J. (2024). Mechanisms of epigallocatechin gallate (EGCG) in ameliorating hyperuricemia: Insights into gut microbiota and intestinal function in a mouse model. Food Funct..

[29] Pan L., Feng R., Hu J., Yu H., Tong Q., Yang X., Song J., Xu H., Ye M., Zhang Z. (2025). Bacteroi des fragilis-derived succinic acid promotes the degradation of uric acid by inhibiting hepatic AMPD2: Insight into how plant-based berberine ameliorates hyperuricemia. Acta Pharm. Sin. B.

[30] Tan W., Xie X., Yu Q., Yan G., Chen H., Xie Q., Liu Y., Huang X., Chen J., Xie J. (2025). Oxyberberine alleviates fructose-induced hyperuricemia by modulating purine metabolism and gut microbiota. Eur. J. Pharmacol..

[31] Liu B.H., Li Z.H., Wang B.R., Zhou J., Zhang B., Wang K.L., Zhang Y.H., Mu Z.S. (2025). Rosmarinic acid in *Perilla frutescens* L. as a potential adenosine deaminase inhibitor: Preparation, machine learning validation and binding mechanism study. Food Chem..

[32] Jia L., Sun B., Nie A., Shi Y., Zhou Z., Zhu C. (2025). Rosmarinic acid attenuates hyperuricemia via restoring hyperuricemia-induced renal and intestinal dysfunctions. Phytomedicine.

[33] Chen Y., Li E.M., Xu L.Y. (2022). Guide to Metabolomics Analysis: A Bioinformatics Workflow. Metabolites.

[34] Cai X., Zhang S., Lan T., Jin Z., Liu J., Jiang Z., Yang Q. (2025). The relationship between dietary index for gut microbiota and hyperuricemia: A cross-sectional study using NHANES data. Front. Nutr..

[35] Liu Y., Li Y.J., Loh Y.W., Singer J., Zhu W., Macia L., Mackay C.R., Wang W., Chadban S.J., Wu H. (2021). Fiber Derived Microbial Metabolites Prevent Acute Kidney Injury Through G-Protein Coupled Receptors and HDAC Inhibition. Front. Cell Dev. Biol..

[36] Li Y., Li L., Tian J., Zheng F., Liao H., Zhao Z., Chen Y., Pang J., Wu T. (2022). Insoluble Fiber in Barley Leaf Attenuates Hyperuricemic Nephropathy by Modulating Gut Microbiota and Short-Chain Fatty Acids. Foods.

[37] Vinelli V., Biscotti P., Martini D., Del Bo C., Marino M., Meroño T., Nikoloudaki O., Calabrese F.M., Turroni S., Taverniti V. (2022). Effects of Dietary Fibers on Short-Chain Fatty Acids and Gut Microbiota Composition in Healthy Adults: A Systematic Review. Nutrients.

[38] Xu X., Wang H., Guo D., Man X., Liu J., Li J., Luo C., Zhang M., Zhen L., Liu X. (2021). Curcumin modulates gut microbiota and improves renal function in rats with uric acid nephropathy. Ren. Fail..

[39] Li C., Chen X., Zha W., Shen J., Lai J., Zheng B., Li L., Jiang H., Tian P. (2026). Curcumin Inhibits Renal Fibrosis by Suppressing S100A8/A9-TLR4 Signaling via Gut Microbiota-Derived Short-Chain Fatty Acid in Macrophages. Mol. Nutr. Food Res..

[40] Xu Y., Cao X., Zhao H., Yang E., Wang Y., Cheng N., Cao W. (2021). Impact of *Camellia japonica* Bee Pollen Polyphenols on Hyperuricemia and Gut Microbiota in Potassium Oxonate-Induced Mice. Nutrients.

[41] Liu Y., Sheng S., Wu L., Wang H., Xue H., Wang R. (2025). Flavonoid-rich extract of *Paederia scandens* (Lour.) Merrill improves hyperuricemia by regulating uric acid metabolism and gut microbiota. Food Chem..

[42] Zhang H., Wang D., Li D., Bao B., Chen Q., Wang S., Han S., Zhao M. (2025). *Lactobacillus paracasei* N1115 alleviates hyperuricemia in mice: Regulation of uric acid metabolism as well as its impact on gut microbiota and short-chain fatty acids. Front. Nutr..

[43] Li Y., Li H., Wang R., Yu Y., Liu X., Tian Z. (2023). Protective effect of sodium butyrate on intestinal barrier damage and uric acid reduction in hyperuricemia mice. Biomed. Pharmacother..

[44] Hung T.V., Suzuki T. (2018). Dietary Fermentable Fibers Attenuate Chronic Kidney Disease in Mice by Protecting the Intestinal Barrier. J. Nutr..

[45] Wu G., Wang X., Dong H., Yu J., Li T., Wang X. (2025). Coix Seed Oil Alleviates Hyperuricemia in Mice by Ameliorating Oxidative Stress and Intestinal Microbial Composition. Nutrients.

[46] Zhang T., Liu S., Liu S., Zhao P., Zhang C., Wang X., Meng Y., Lu Y. (2025). Oleanolic Acid Alleviates Hyperuricemia via Gut Microbiota Control the Integrity of Gut Barrier and the Expressions of Urate Transporter in Mice. J. Agric. Food Chem..

[47] Ren P., Wei B., Qin W., Tang Q., Wang Y., Xue C. (2025). Impact of astaxanthin on the capacity of gut microbiota to produce tryptophan catabolites. Food Funct..

[48] Ni S., Zheng W., Hu Y., Li S., Song S., Ai C. (2025). Fabrication and characterization of astaxanthin-loaded nanoprobiotic and its role in alleviating hyperuricemia via the regulation of the gut-kidney axis. Int. J. Biol. Macromol..

[49] Song X., Sun X., Oh S.F., Wu M., Zhang Y., Zheng W., Geva-Zatorsky N., Jupp R., Mathis D., Benoist C. (2020). Microbial bile acid metabolites modulate gut RORγ^+^ regulatory T cell homeostasis. Nature.

[50] Gan Y., Zeng Y., Huang J., Li Y., Zhu Q., Wang L. (2025). Polysaccharide extracted from *Phellinus igniarius* attenuated hyperuricemia by modulating bile acid metabolism and inhibiting uric acid synthesis in adenine/potassium oxonate-treated mice. J. Ethnopharmacol..

[51] Zou T., Zhang R., Chen Z., Duan H., Wang Z., Gao R., Chen X., Wang H., Zhao Y., Wang X. (2025). Sustained hyperuricemia disrupts enterohepatic circulation through 12α-hydroxy BA and gut microbiota. Front. Microbiol..

[52] Arifuzzaman M., Won T.H., Li T.T., Yano H., Digumarthi S., Heras A.F., Zhang W., Parkhurst C.N., Kashyap S., Jin W.B. (2022). Inulin fibre promotes microbiota-derived bile acids and type 2 inflammation. Nature.

[53] Wang S., Chen J., Li Y., Wang Y. (2025). Secoisolariciresinol diglucoside (SDG) from flaxseed meal alleviates hyperuricemia in mice by regulating uric acid metabolism and intestinal homeostasis. Food Res. Int..

[54] Leng Y., Xie T., Tao Y., Zeng Y., Zhu Q., Wang L. (2025). Roles of an endogenous peptide ELABELA in ameliorating hyperuricemia by inhibiting uric acid production via CYP27A1-modulated bile acid metabolism in mice. Biochem. Pharmacol..

[55] Bao R., Wang W., Chen B., Pan J., Chen Q., Liu M., Wang D., Wu Y., Yu H., Han L. (2022). Dioscin Ameliorates Hyperuricemia-Induced Atherosclerosis by Modulating of Cholesterol Metabolism through FXR-Signaling Pathway. Nutrients.

[56] Wu J., Aga L., Tang L., Li H., Wang N., Yang L., Zhang N., Wang X., Wang X. (2024). *Lacticaseibacillus paracasei* JS-3 Isolated from “Jiangshui” Ameliorates Hyperuricemia by Regulating Gut Microbiota and iTS Metabolism. Foods.

[57] Gupta B., Liu Y., Chopyk D.M., Rai R.P., Desai C., Kumar P., Farris A.B., Nusrat A., Parkos C.A., Anania F.A. (2020). Western diet-induced increase in colonic bile acids compromises epithelial barrier in nonalcoholic steatohepatitis. FASEB J. Off. Publ. Fed. Am. Soc. Exp. Biol..

[58] Liu C., Ruan F., Chen Z., Han J., Ding X., Han C., Ye L., Yang C., Yu Y., Zuo Z. (2024). Phenanthrene-induced hyperuricemia with intestinal barrier damage and the protective role of theabrownin: Modulation by gut microbiota-mediated bile acid metabolism. Sci. Total Environ..

[59] Zhou X., Zhang B., Zhao X., Lin Y., Zhuang Y., Guo J., Wang S. (2022). Chlorogenic Acid Prevents Hyperuricemia Nephropathy via Regulating TMAO-Related Gut Microbes and Inhibiting the PI3K/AKT/mTOR Pathway. J. Agric. Food Chem..

[60] Graboski A.L., Redinbo M.R. (2020). Gut-Derived Protein-Bound Uremic Toxins. Toxins.

[61] Lauriola M., Farré R., Evenepoel P., Overbeek S.A., Meijers B. (2023). Food-Derived Uremic Toxins in Chronic Kidney Disease. Toxins.

[62] Guo Y., Yu Y., Li H., Ding X., Li X., Jing X., Chen J., Liu G., Lin Y., Jiang C. (2021). Inulin supplementation ameliorates hyperuricemia and modulates gut microbiota in Uox-knockout mice. Eur. J. Nutr..

[63] Xia J., Zhang Y., Zhang S., Lu C., Huan H., Guan X. (2024). Oat Dietary Fiber Delays the Progression of Chronic Kidney Disease in Mice by Modulating the Gut Microbiota and Reducing Uremic Toxin Levels. J. Agric. Food Chem..

[64] Peng B., Dai J., Ji S., Yang Y., Zuo A., Xu S., Fang W., Li D., You Y., Jiang Z. (2025). Quercetin ameliorates hyperuricemic nephropathy through improving gut dysfunctions and decreasing gut bacteria-derived uremic toxins. Phytomed. Int. J. Phytother. Phytopharm..

[65] Xie J., Ma X., Zheng Y., Mao N., Ren S., Fan J. (2022). Panax notoginseng saponins alleviate damage to the intestinal barrier and regulate levels of intestinal microbes in a rat model of chronic kidney disease. Ren. Fail..

[66] Nigam S.K., Granados J.C. (2023). OAT, OATP, and MRP Drug Transporters and the Remote Sensing and Signaling Theory. Annu. Rev. Pharmacol. Toxicol..

[67] Han R., Wang Z., Li Y., Ke L., Li X., Li C., Tian Z., Liu X. (2026). Gut microbiota *Lactobacillus johnsonii* alleviates hyperuricemia by modulating intestinal urate and gut microbiota-derived butyrate. Chin. Med. J..

[68] Wei B., Ren P., Yang R., Gao Y., Tang Q., Xue C., Wang Y. (2023). Ameliorative Effect of Mannuronate Oligosaccharides on Hyperuricemic Mice via Promoting Uric Acid Excretion and Modulating Gut Microbiota. Nutrients.

[69] Luo J.J., Chen X.H., Liang P.Y., Zhao Z., Wu T., Li Z.H., Wan S.H., Luo J., Pang J.X., Zhang J.J. (2023). Mechanism of anti-hyperuricemia of isobavachin based on network pharmacology and molecular docking. Comput. Biol. Med..

[70] Canyelles M., Borràs C., Rotllan N., Tondo M., Escolà-Gil J.C., Blanco-Vaca F. (2023). Gut Microbiota-Derived TMAO: A Causal Factor Promoting Atherosclerotic Cardiovascular Disease?. Int. J. Mol. Sci..

[71] Yanai H., Adachi H., Hakoshima M., Iida S., Katsuyama H. (2024). A Possible Therapeutic Application of the Selective Inhibitor of Urate Transporter 1, Dotinurad, for Metabolic Syndrome, Chronic Kidney Disease, and Cardiovascular Disease. Cells.

[72] Ermakov V.S., Granados J.C., Nigam S.K. (2023). Remote effects of kidney drug transporter OAT1 on gut microbiome composition and urate homeostasis. JCI Insight.

[73] Zhao T., Cao L., Lin C., Xu R., Du X., Zhou M., Yang X., Wan W., Zou H., Zhu X. (2023). Intestinal uric acid excretion contributes to serum uric acid decrease during acute gout attack. Rheumatology.

[74] Li T.T., Chen X., Huo D., Arifuzzaman M., Qiao S., Jin W.B., Shi H., Li X.V., Iliev I.D., Artis D. (2024). Microbiota metabolism of intestinal amino acids impacts host nutrient homeostasis and physiology. Cell Host Microbe.

[75] Wu D., Niu J., Hu J., Wang H., Kuang H. (2025). Metabolomics combined with metagenomics analysis reveals the potential mechanism of *Zhejiang psyllium* polysaccharides against hyperuricemia in rats. Sci. Rep..

[76] Wu D., Li Z., Zhang Y., Zhang Y., Ren G., Zeng Y., Liu H., Guan W., Zhao X., Li P. (2023). Proline uptake promotes activation of lymphoid tissue inducer cells to maintain gut homeostasis. Nat. Metab..

[77] Liu P., Hu P., Jin M., Sun W., Wu J., Tang Y., Shi D., Xie T., Tong Y., Huang L. (2025). Compound probiotics alleviate hyperuricemia-induced renal injury via restoring gut microbiota and metabolism. BMC Microbiol..

[78] Liu X., Zhao F., Liu H., Xie Y., Zhao D., Li C. (2021). Transcriptomics and metabolomics reveal the adaption of *Akkermansia muciniphila* to high mucin by regulating energy homeostasis. Sci. Rep..

[79] Tobón-Cornejo S., Sanchez-Tapia M., Guizar-Heredia R., Velázquez Villegas L., Noriega L.G., Furuzawa-Carballeda J., Hernández-Pando R., Vázquez-Manjarrez N., Granados-Portillo O., López-Barradas A. (2025). Increased dietary protein stimulates amino acid catabolism via the gut microbiota and secondary bile acid production. Gut Microbes.

[80] Zhan X., He M., Pei J., Fan W., Mwangi C.N., Zhang P., Chai X., Jiang M. (2022). Natural Phenylethanoid Supplementation Alleviates Metabolic Syndrome in Female Mice Induced by High-Fructose Diet. Front. Pharmacol..

[81] Yang X., Li H., Qumu D., Han B., Amatjan M., Wu Q., Wei L., Li B., Ma M., He J. (2025). Taurine alleviates hyperuricemia-induced nephropathy in rats: Insights from microbiome and metabolomics. Front. Nutr..

[82] Zhang Q.Z., Zhang J.R., Li X., Yin J.L., Jin L.M., Xun Z.R., Xue H., Yang W.Q., Zhang H., Qu J. (2024). Fangyukangsuan granules ameliorate hyperuricemia and modulate gut microbiota in rats. Front. Immunol..

[83] Li J., Song L., Liang X., Zhou J., Luo J., Sun X., Jin R., Zhang Z. (2026). *Lactobacillus plantarum* TY-S8 ameliorates hyperuricemia through the regulation of gut microbiota and tryptophan metabolism in mice. Food Funct..

[84] Wang Q., Liang J., Zou Q., Wang W., Yan G., Guo R., Yuan T., Wang Y., Liu X., Liu Z. (2024). Tryptophan Metabolism-Regulating Probiotics Alleviate Hyperuricemia by Protecting the Gut Barrier Integrity and Enhancing Colonic Uric Acid Excretion. J. Agric. Food Chem..

[85] Oshima S., Shiiya S., Nakamura Y. (2019). Combined Supplementation with Glycine and Tryptophan Reduces Purine-Induced Serum Uric Acid Elevation by Accelerating Urinary Uric Acid Excretion: A Randomized, Single-Blind, Placebo-Controlled, Crossover Study. Nutrients.

[86] Wang Y., Miao F., Wang J., Zheng M., Yu F., Yi Y. (2024). The ameliorative and neuroprotective effects of dietary fibre on hyperuricaemia mice: A perspective from microbiome and metabolome. Br. J. Nutr..

[87] Tan X., Gao B., Xu Y., Zhao Q., Jiang J., Sun D., Zhang Y., Zhou S., Fan J.B., Zhang M. (2025). Atractylodes macrocephala-derived extracellular vesicles-like particles enhance the recovery of ulcerative colitis by remodeling intestinal microecological balance. J. Nanobiotechnol..

[88] Zeng Q., Li D., He Y., Li Y., Yang Z., Zhao X., Liu Y., Wang Y., Sun J., Feng X. (2019). Discrepant gut microbiota markers for the classification of obesity-related metabolic abnormalities. Sci. Rep..

[89] Xu H., Fang F., Wu K., Song J., Li Y., Lu X., Liu J., Zhou L., Yu W., Yu F. (2023). Gut microbiota-bile acid crosstalk regulates murine lipid metabolism via the intestinal FXR-FGF19 axis in diet-induced humanized dyslipidemia. Microbiome.

[90] Xu M., Xiao H., Zou X., Pan L., Song Q., Hou L., Zeng Y., Han Y., Zhou Z. (2025). Mechanisms of levan in ameliorating hyperuricemia: Insight into levan on serum metabolites, gut microbiota, and function in hyperuricemia rats. Carbohydr. Polym..

[91] Liu X., Ma L., Fan Z., Fan J., Lu W., Zhang L., Li J. (2026). Protein hydrolysates and xanthine oxidase inhibitory peptides from sunflower capitulum exert antihyperuricemic effects: A comprehensive study based on serum metabolomics and gut microbiota analysis. Food Funct..

[92] Liu X., Feng Z., Zhang F., Wang B., Wei Z., Liao N., Zhang M., Liang J., Wang L. (2024). Causal effects of gut microbiota on gout and hyperuricemia: Insights from genome-wide Mendelian randomization, RNA-sequencing, 16S rRNA sequencing, and metabolomes. Biosci. Rep..

[93] Ji X., Yu L., Han C., Gao H., Cai Y., Li J., He Y., Lu H., Song G., Xue P. (2024). Investigating the effects of rare ginsenosides on hyperuricemia and associated sperm damage via nontargeted metabolomics and gut microbiota. J. Ethnopharmacol..

[94] Waldman B., Ansquer J.C., Sullivan D.R., Jenkins A.J., McGill N., Buizen L., Davis T.M.E., Best J.D., Li L., Feher M.D. (2018). Effect of fenofibrate on uric acid and gout in type 2 diabetes: A post-hoc analysis of the randomised, controlled FIELD study. Lancet Diabetes Endocrinol..

[95] Shi M., Lu Y., Wu J., Zheng Z., Lv C., Ye J., Qin S., Zeng C. (2022). Beneficial Effects of Theaflavins on Metabolic Syndrome: From Molecular Evidence to Gut Microbiome. Int. J. Mol. Sci..

[96] Yang H., Liang Y., Chen Y., Liu L., Wang Y., Dou Q., Gong J., Liao Z., Dong J., Hu X. (2026). The mechanism of honeysuckle peptides in ameliorating hyperuricemia in mice via the PGC-1α/PPARγ/ABCG2 pathway. J. Ethnopharmacol..

[97] Yang Y., Wang Y., Huang J., Xu Y., Yin X., Lin Z., Zhang B. (2025). Influence of Gut Microbiota-Derived Butyrate on Intestinal Uric Acid Excretion and Hyperuricemia Regulation by *Cichorium intybus* L.. Int. J. Mol. Sci..

[98] Xia X., Niu H., Xu C., Liu X., Zhang G., Ling J. (2026). Revisiting the metabolic crosstalk between type 2 diabetes and hyperuricemia: Pathophysiological insights and therapeutic perspectives. Biochim. Biophys. Acta Mol. Basis Dis..

[99] Chen Z., Liu J., Ding H., Yan C., Zhu H., Huang S., Chen Z.Y. (2024). Dietary supplementation with capsaicinoids alleviates obesity in mice fed a high-fat-high-fructose diet. Food Funct..

[100] He L., Miao M., Li Q., Cheng J., Li R. (2025). Evaluation of the Effects of High Uric Acid on Glucolipid Metabolism, Renal Injury and the Gut Microbiota in Diabetic Male Hamsters with Dyslipidemia. Toxics.

[101] Li Q., Hu J., Nie Q., Chang X., Fang Q., Xie J., Li H., Nie S. (2021). Hypoglycemic mechanism of polysaccharide from *Cyclocarya paliurus* leaves in type 2 diabetic rats by gut microbiota and host metabolism alteration. Sci. China Life Sci..

[102] Lin H., Nie L., Wu D., Zhang D., Peng R., Tao S., Ye Z., Zhu S., Ye M., Xiao J. (2025). Cathepsin B-dependent glycolysis contributes to reduced renal uric acid excretion in hyperuricemia. Commun. Biol..

[103] Han Q.Q., Ren Q.D., Guo X., Farag M.A., Zhang Y.H., Zhang M.Q., Chen Y.Y., Sun S.T., Sun J.Y., Li N.Y. (2025). Punicalagin attenuates hyperuricemia via restoring hyperuricemia-induced renal and intestinal dysfunctions. J. Adv. Res..

[104] Wang Z., Zhang Z., Lu C., Zhou J., Wang Z., Han J., Su X. (2022). Effects of *Sporisorium reiliana* polysaccharides and Phoenix dactylifera monosaccharides on the gut microbiota and serum metabolism in mice with fructose-induced hyperuricemia. Arch. Microbiol..

[105] Renaudin F., Orliaguet L., Castelli F., Fenaille F., Prignon A., Alzaid F., Combes C., Delvaux A., Adimy Y., Cohen-Solal M. (2020). Gout and pseudo-gout-related crystals promote GLUT1-mediated glycolysis that governs NLRP3 and interleukin-1β activation on macrophages. Ann. Rheum. Dis..

[106] Krautkramer K.A., Fan J., Bäckhed F. (2021). Gut microbial metabolites as multi-kingdom intermediates. Nat. Rev. Microbiol..

[107] Tulkens J., Vergauwen G., Van Deun J., Geeurickx E., Dhondt B., Lippens L., De Scheerder M.A., Miinalainen I., Rappu P., De Geest B.G. (2020). Increased levels of systemic LPS-positive bacterial extracellular vesicles in patients with intestinal barrier dysfunction. Gut.

[108] Di Vincenzo F., Del Gaudio A., Petito V., Lopetuso L.R., Scaldaferri F. (2024). Gut microbiota, intestinal permeability, and systemic inflammation: A narrative review. Intern. Emerg. Med..

[109] Mann E.R., Lam Y.K., Uhlig H.H. (2024). Short-chain fatty acids: Linking diet, the microbiome and immunity. Nat. Rev. Immunol..

[110] Vieira A.T., Galvão I., Macia L.M., Sernaglia É.M., Vinolo M.A., Garcia C.C., Tavares L.P., Amaral F.A., Sousa L.P., Martins F.S. (2017). Dietary fiber and the short-chain fatty acid acetate promote resolution of neutrophilic inflammation in a model of gout in mice. J. Leukoc. Biol..

[111] Xi Y., Huang Y., Li Y., Huang Y., Yan J., Shi Z. (2022). The effects of dietary protein and fiber levels on growth performance, gout occurrence, intestinal microbial communities, and immunoregulation in the gut-kidney axis of goslings. Poult. Sci..

[112] Fan S., Huang Y., Lu G., Sun N., Wang R., Lu C., Ding L., Han J., Zhou J., Li Y. (2022). Novel anti-hyperuricemic hexapeptides derived from *Apostichopus japonicus* hydrolysate and their modulation effects on the gut microbiota and host microRNA profile. Food Funct..

[113] Si K., Zhang W., Qi C., Chi J., Xu L., Wang Y., Chen Y., Wang W., Xue Y., Wang Y. (2026). Regulatory role and mechanism of the probiotics on monosodium urate crystal-induced gout inflammation. Clin. Rheumatol..

[114] Wang Q., Hu Z., Wang Y., Guo S., Zhang X., Ren Y., Li J., Li X., Cui H. (2026). *Lactobacillus paragasseri* LG-1 Alleviates Urticaria-Like Symptoms in Mice via Modulation of Gut Microbiota, Hypoxanthine and Uric Acid. Microb. Biotechnol..

[115] Li Y., Han R., Wang R., Zhang X., Shan M., Fu T., Li Y., Qi X., Cao B., Wang Y. (2026). Alginate alleviates hyperuricemia by modulating the *Pediococcus acidilactici* LW1-1-butyrate-NLRP3 inflammasome axis. Int. J. Biol. Macromol..

[116] Wu H., Pang M.M., Li Y.L., Hong J.H., Liu P.M., Bian M., Yang J.J. (2025). Allicin Aplealleviates Gouty Arthritis by Regulating the Gut-Joint Axis, Reducing XOD Activity, Inhibiting Oxidative Stress, and Suppressing NLRP3 Inflammasome Activation. Drug Des. Dev. Ther..

[117] Han J., Wang Z., Lu C., Zhou J., Li Y., Ming T., Zhang Z., Wang Z.J., Su X. (2021). The gut microbiota mediates the protective effects of anserine supplementation on hyperuricaemia and associated renal inflammation. Food Funct..

[118] Sun Q., Li Z., Yu Y., Sun Y. (2025). Mechanism of Ginsenoside Rg(1) in Regulating the Metabolic Function of Intestinal Flora for the Treatment of High-Purine Dietary Hyperuricemia. Nutrients.

[119] Wu Y.L., Chen J.F., Jiang L.Y., Wu X.L., Liu Y.H., Gao C.J., Wu Y., Yi X.Q., Su Z.R., Cai J. (2021). The Extract of Sonneratia apetala Leaves and Branches Ameliorates Hyperuricemia in Mice by Regulating Renal Uric Acid Transporters and Suppressing the Activation of the JAK/STAT Signaling Pathway. Front. Pharmacol..

[120] Song S., Shi K., Fan M., Wen X., Li J., Guo Y., Lou Y., Chen F., Wang J., Huang L. (2026). Clostridium butyricum and its metabolites regulate macrophage polarization through miR-146a to antagonize gouty arthritis. J. Adv. Res..

[121] Tian Z., Zhang J., Tan Z., Duan X., Zhang H., Li B., Wang E., Wang V.Y., Xu D., Ma J. (2025). *Lactiplantibacillus plantarum* MPB-65 combined with epicatechin alleviates hyperuricemia by modulating the gut-kidney axis. Food Funct..

[122] Wang Q., Pu X., Song Y., Lv Z., Hao J., Yuan T., Wang Y., Liu X., Guo R., Liu Z. (2025). SCFAs-Enriching Kiwifruit-Derived Synbiotic Reprograms Microbiota to Suppress XOD and Promote Urate Clearance. J. Agric. Food Chem..

[123] Nie D., Niu M., Wang X., Liu X., Zhang Z., Zhou Y., Liu C. (2026). Identification of the polyphenols from Lonicera flos-Lonicera Caulis compatibility in gut content and their implication in ameliorating gouty arthritis. Fitoterapia.

[124] Xiao Y., Zhang T., Chen Q., Zhang Y., Chen B., Wang M., Zhang Y., Huang M., Su Y., Guo J. (2026). Multi-omics analysis reveals the mechanism of verbenalin in treating gout via modulating purine metabolism, gut microbiota, and inflammatory pathways. Front. Immunol..

[125] Ribeiro A., Liu F., Srebrzynski M., Rother S., Adamowicz K., Wadowska M., Steiger S., Anders H.J., Schmaderer C., Koziel J. (2023). Uremic Toxin Indoxyl Sulfate Promotes Macrophage-Associated Low-Grade Inflammation and Epithelial Cell Senescence. Int. J. Mol. Sci..

[126] Shi G., Zeng L., Shi J., Chen Y. (2023). Trimethylamine N-oxide Promotes Atherosclerosis by Regulating Low-Density Lipoprotein-Induced Autophagy in Vascular Smooth Muscle Cells Through PI3K/AKT/mTOR Pathway. Int. Heart J..

[127] Zhou X., Zhang B., Zhao X., Lin Y., Wang J., Wang X., Hu N., Wang S. (2021). Chlorogenic acid supplementation ameliorates hyperuricemia, relieves renal inflammation, and modulates intestinal homeostasis. Food Funct..

[128] Qie J., Cao M., Xu M., Zhang Y., Luo L., Sun C., Ke D., Yuan S., Jia W., Qiu T. (2025). Multi-cohort analysis unveils novel microbial targets for the treatment of hyperuricemia and gout. mSystems.

[129] Kim H.W., Yoon E.J., Jeong S.H., Park M.C. (2022). Distinct Gut Microbiota in Patients with Asymptomatic Hyperuricemia: A Potential Protector against Gout Development. Yonsei Med. J..

[130] Martínez-Nava G.A., Méndez-Salazar E.O., Vázquez-Mellado J., Zamudio-Cuevas Y., Francisco-Balderas A., Martínez-Flores K., Fernández-Torres J., Lozada-Pérez C., Guido-Gómora D.L., Martínez-Gómez L.E. (2023). The impact of short-chain fatty acid-producing bacteria of the gut microbiota in hyperuricemia and gout diagnosis. Clin. Rheumatol..

[131] Chu Y., Sun S., Huang Y., Gao Q., Xie X., Wang P., Li J., Liang L., He X., Jiang Y. (2021). Metagenomic analysis revealed the potential role of gut microbiome in gout. NPJ Biofilms Microbiomes.

[132] Zeng Y., Guo M., Fang X., Teng F., Tan X., Li X., Wang M., Long Y., Xu Y. (2021). Gut Microbiota-Derived Trimethylamine N-Oxide and Kidney Function: A Systematic Review and Meta-Analysis. Adv. Nutr..

[133] Hsu C.N., Lu P.C., Lo M.H., Lin I.C., Chang-Chien G.P., Lin S., Tain Y.L. (2018). Gut Microbiota-Dependent Trimethylamine N-Oxide Pathway Associated with Cardiovascular Risk in Children with Early-Stage Chronic Kidney Disease. Int. J. Mol. Sci..

[134] Gryp T., Huys G.R.B., Joossens M., Van Biesen W., Glorieux G., Vaneechoutte M. (2020). Isolation and Quantification of Uremic Toxin Precursor-Generating Gut Bacteria in Chronic Kidney Disease Patients. Int. J. Mol. Sci..

[135] Yang H.L., Feng P., Xu Y., Hou Y.Y., Ojo O., Wang X.H. (2021). The Role of Dietary Fiber Supplementation in Regulating Uremic Toxins in Patients With Chronic Kidney Disease: A Meta-Analysis of Randomized Controlled Trials. J. Ren. Nutr..

[136] Khosroshahi H.T., Abedi B., Ghojazadeh M., Samadi A., Jouyban A. (2019). Effects of fermentable high fiber diet supplementation on gut derived and conventional nitrogenous product in patients on maintenance hemodialysis: A randomized controlled trial. Nutr. Metab..

[137] Li T., Lu Z., Peng W., Liu J., Yuan J., Zhu L., Zhou Y., Yang C., Zhu Y. (2026). Oatmeal-based fiber diet outperforms resistant starch-based fiber diet in lowering serum uric acid via gut microbiota-metabolite interactions: A randomized controlled trial. Food Funct..

[138] Xing R., Fan K., Fan Z., Wang L., Huang Y., Zhang H., Chen W., Si X. (2025). Porcine bile acids improve performance by altering hepatic lipid metabolism and amino acid metabolism with different protein level diets in late laying hens. Poult. Sci..

[139] Lai S., Molfino A., Testorio M., Perrotta A.M., Currado A., Pintus G., Pietrucci D., Unida V., La Rocca D., Biocca S. (2019). Effect of Low-Protein Diet and Inulin on Microbiota and Clinical Parameters in Patients with Chronic Kidney Disease. Nutrients.

[140] Zhuo L.B., Yang Y., Xiao C., Li F., Lin L., Xi Y., Fu Y., Zheng J.S., Chen Y.M. (2024). Gut microbiota-bile acid axis mediated the beneficial associations between dietary lignans and hyperuricemia: A prospective study. Food Funct..

[141] Zhang F., Lim W.L.F., Huang Y., Lam S.M., Wang Y. (2024). Lipidomics and metabolomics investigation into the effect of DAG dietary intervention on hyperuricemia in athletes. J. Lipid Res..

[142] Pivari F., Mingione A., Piazzini G., Ceccarani C., Ottaviano E., Brasacchio C., Dei Cas M., Vischi M., Cozzolino M.G., Fogagnolo P. (2022). Curcumin Supplementation (Meriva^®^) Modulates Inflammation, Lipid Peroxidation and Gut Microbiota Composition in Chronic Kidney Disease. Nutrients.

[143] Rossi M., Johnson D.W., Morrison M., Pascoe E.M., Coombes J.S., Forbes J.M., Szeto C.C., McWhinney B.C., Ungerer J.P., Campbell K.L. (2016). Synbiotics Easing Renal Failure by Improving Gut Microbiology (SYNERGY): A Randomized Trial. Clin. J. Am. Soc. Nephrol..

[144] Jia L., Jia Q., Yang J., Jia R., Zhang H. (2018). Efficacy of Probiotics Supplementation On Chronic Kidney Disease: A Systematic Review and Meta-Analysis. Kidney Blood Press. Res..

[145] Zhao F., Tie N., Kwok L.Y., Ma T., Wang J., Man D., Yuan X., Li H., Pang L., Shi H. (2024). Baseline gut microbiome as a predictive biomarker of response to probiotic adjuvant treatment in gout management. Pharmacol. Res..

[146] Yuan S., Jia W., Liu X., Liu R., Cao M., Wu Y., Li Y., Xu W., Xiao C., Hong Z. (2025). Therapeutic effect of fecal microbiota transplantation on hyperuricemia mice by improving gut microbiota. Front. Microbiol..

[147] Yu Y., Wang W., Zhang F. (2023). The Next Generation Fecal Microbiota Transplantation: To Transplant Bacteria or Virome. Adv. Sci..

[148] Xie W.R., Yang X.Y., Deng Z.H., Zheng Y.M., Zhang R., Wu L.H., Cai J.Y., Kong L.P., Xia H.H., He X.X. (2022). Effects of Washed Microbiota Transplantation on Serum Uric Acid Levels, Symptoms, and Intestinal Barrier Function in Patients with Acute and Recurrent Gout: A Pilot Study. Dig. Dis..

[149] Deng X., Zhang C., Wang P., Wei W., Shi X., Wang P., Yang J., Wang L., Tang S., Fang Y. (2022). Cardiovascular Benefits of Empagliflozin Are Associated With Gut Microbiota and Plasma Metabolites in Type 2 Diabetes. J. Clin. Endocrinol. Metab..

[150] Kavyani M., Saleh-Ghadimi S., Dehghan P., Abbasalizad Farhangi M., Khoshbaten M. (2021). Co-supplementation of camelina oil and a prebiotic is more effective for in improving cardiometabolic risk factors and mental health in patients with NAFLD: A randomized clinical trial. Food Funct..

[151] Lao B.N., Luo J.H., Xu X.Y., Fu L.Z., Tang F., Ouyang W.W., Xu X.Z., Wei M.T., Xiao B.J., Chen L.Y. (2023). Time-restricted feeding’s effect on overweight and obese patients with chronic kidney disease stages 3-4: A prospective non-randomized control pilot study. Front. Endocrinol..

[152] Lin J.H., Lin C.H., Kuo Y.W., Liao C.A., Chen J.F., Tsai S.Y., Li C.M., Hsu Y.C., Huang Y.Y., Hsia K.C. (2024). Probiotic *Lactobacillus fermentum* TSF331, *Lactobacillus reuteri* TSR332, and *Lactobacillus plantarum* TSP05 improved liver function and uric acid management-A pilot study. PLoS ONE.

[153] Ding D., Yong H., You N., Lu W., Yang X., Ye X., Wang Y., Cai T., Zheng X., Chen H. (2022). Prospective Study Reveals Host Microbial Determinants of Clinical Response to Fecal Microbiota Transplant Therapy in Type 2 Diabetes Patients. Front. Cell. Infect. Microbiol..

[154] Cao C., Li F., Ding Q., Jin X., Tu W., Zhu H., Sun M., Zhu J., Yang D., Fan B. (2024). Potassium sodium hydrogen citrate intervention on gut microbiota and clinical features in uric acid stone patients. Appl. Microbiol. Biotechnol..

[155] Kondratiuk V.E., Tarasenko O.M., Karmazina O.M., Taranchuk V.V. (2020). Impact of the Synbiotics and Urate-Lowering Therapy on Gut Microbiota and Cytokine Profile in Patients with Chronic Gouty Arthritis. J. Med. Life.

[156] Kalidindi R.K., Reddy C.P., Pv K., Kompella P. (2024). The Efficacy and Safety of Probiotic Combinations Lobun Forte^®^ Versus Renadyl^®^ in Patients With Chronic Kidney Disease: A Comparative, Phase IV, Randomized, Open-Label, Active-Controlled, Parallel Study. Cureus.

[157] Madella A.M., Van Bergenhenegouwen J., Garssen J., Masereeuw R., Overbeek S.A. (2022). Microbial-Derived Tryptophan Catabolites, Kidney Disease and Gut Inflammation. Toxins.

[158] Li Y., Taherkhani S., Saidov K., Matniyozova M. (2025). Integrative multi-omics profiling for early diagnosis, stratification and personalized management of chronic kidney disease: A new paradigm. Clin. Exp. Med..

[159] Bharti R., Grimm D.G. (2021). Current challenges and best-practice protocols for microbiome analysis. Brief. Bioinform..

[160] Carr A.V., Baliga N.S., Diener C., Gibbons S.M. (2025). Personalized Clostridioides difficile colonization risk prediction and probiotic therapy assessment in the human gut. Cell Syst..

[161] Costea P.I., Hildebrand F., Arumugam M., Bäckhed F., Blaser M.J., Bushman F.D., de Vos W.M., Ehrlich S.D., Fraser C.M., Hattori M. (2018). Enterotypes in the landscape of gut microbial community composition. Nat. Microbiol..

[162] Xiao P., Li Y., Li X., Ge T., Li D., Xu Q., Ruan Y., Xiao F., Xiao Y., Zhang T. (2025). Long-term safety of fecal microbiota transplantation in Chinese children from 2013 to 2023: A single-center retrospective study. BMC Microbiol..

[163] Merenstein D., Pot B., Leyer G., Ouwehand A.C., Preidis G.A., Elkins C.A., Hill C., Lewis Z.T., Shane A.L., Zmora N. (2023). Emerging issues in probiotic safety: 2023 perspectives. Gut Microbes.

[164] Zhang N.N., Jiang Z.M., Li S.Z., Yang X., Liu E.H. (2023). Evolving interplay between natural products and gut microbiota. Eur. J. Pharmacol..

[165] Myhill L.J., Jensen P., Arora P., Jensen A.M., Zhu L., Vedsted-Jakobsen A., Thormar E.A., von Münchow A., Poojary M.M., Lund M.N. (2026). Dietary fibre promotes chronic gut parasite infection via direct and time-dependent modulation of innate immunity. Microbiome.

[166] Chen P., Chen F., Hou T., Hu X., Xia C., Zhang J., Shen S., Li C., Li K. (2025). Administration time modify the anxiolytic and antidepressant effects of inulin via gut-brain axis. Int. J. Biol. Macromol..

[167] Li H., Li M., Fu J., Ao H., Wang W., Wang X. (2021). Enhancement of oral bioavailability of quercetin by metabolic inhibitory nanosuspensions compared to conventional nanosuspensions. Drug Deliv..

[168] Andres S., Pevny S., Ziegenhagen R., Bakhiya N., Schäfer B., Hirsch-Ernst K.I., Lampen A. (2018). Safety Aspects of the Use of Quercetin as a Dietary Supplement. Mol. Nutr. Food Res..

[169] Ali N. (2026). Interactions between serum uric acid and gut microbiota: Implications for metabolic health. Metab. Open.

[170] Cui Y., Sun W., Wei L., Fan S., Li Q., Duan L. (2026). Complex interactions of gut-derived short-chain fatty acids in hyperuricemia and gout pathophysiology. Front. Microbiol..

[171] Salvi P.S., Cowles R.A. (2021). Butyrate and the Intestinal Epithelium: Modulation of Proliferation and Inflammation in Homeostasis and Disease. Cells.

[172] Hays K.E., Pfaffinger J.M., Ryznar R. (2024). The interplay between gut microbiota, short-chain fatty acids, and implications for host health and disease. Gut Microbes.

[173] Winston J.A., Theriot C.M. (2020). Diversification of host bile acids by members of the gut microbiota. Gut Microbes.

